# Just beautiful green herbs: use of plants in cultural practices in Bukovina and Roztochya, Western Ukraine

**DOI:** 10.1186/s13002-021-00439-y

**Published:** 2021-03-04

**Authors:** N. Stryamets, M. F. Fontefrancesco, G. Mattalia, J. Prakofjewa, A. Pieroni, R. Kalle, G. Stryamets, R. Sõukand

**Affiliations:** 1 Ca’ Foscari University of Venice, Via Torino, 155 Venice, VE Italy; 2 University of Gastronomic Sciences, Piazza Vittorio Emanuele 9, 12042, Bra, Pollenzo, CN Italy; 3 Durham University, South Rd, DH1 3LE Durham, UK; 4 Medical Analysis Department, Tishk International University, Erbil, Kurdistan Region 44001 Iraq; 5 Nature reserve “Roztochya”, Sitchovyh Strilciv 7, Ivano-Frankove, Lviv region 81070 Ukraine

**Keywords:** Ceremonies, Forest products, Hutsuls, Non-wood forest products, Religious rituals, TEK, Biocultural diversity

## Abstract

**Background:**

The use of plants in rituals is a little explored corner of biocultural diversity which has developed through time within a complex socio-ecological system. Indeed, rituals are complex interactions between humans and biodiversity shaped by history, culture, and ethnic belonging. Yet, in Western Ukraine, such rituals were forbidden for over 50 years (1939–1991). The current revival of rituals by rural inhabitants is an untapped reservoir of local ecological knowledge. The aim of the present study was to identify the ritual use of wild and cultivated plants in two regions of Western Ukraine, Bukovina and Roztochya, and to compare the findings with historical data. Moreover, we analyzed attitudes toward the ritual use of plants and interactions with the local environment.

**Methods:**

We conducted 31 in-depth semi-structured interviews among Orthodox Hutsuls of Bukovina and 16 interviews among Greek Catholic rural inhabitants of Roztochya during summer 2018 focusing on the ritual uses of plants.

**Results:**

We documented  28 plant taxa among Bukovinian Hutsuls and 58 plant taxa among inhabitants in Roztochya that were used in 7 religious festivals (of which two were celebrated differently in the two communities). Plants were mainly used in bouquets, but also for decorating churches and houses or in fruit baskets. In both communities, almost 25% of the interviewees could not name the plants they collected for bouquets, but rather referred to “*just beautiful green herbs*” one can get in meadows, forests, and gardens. Comparison with historical data shows a smaller number of taxa currently used (wild taxa have been lost), yet the persistence of 18 taxa used both now and a century ago.

**Conclusions:**

Contemporary practices concerning the use of plants in Christian rituals in Bukovina and Roztochya can be contextualized in the broader phenomenon of the revitalization of traditional environmental knowledge and practices that have characterized Europe over the past 30 years and in particular Eastern Europe after socialism. The current religious use of plants is to a certain extent the revitalization of historical rituals supported by various internal (knowledge from older generations) and external (church authorities and fashion in the region) drivers. Further research should address changes in regions with longer and more severe prohibition of religious practices and their revival.

## Background

**Fig. 1:**
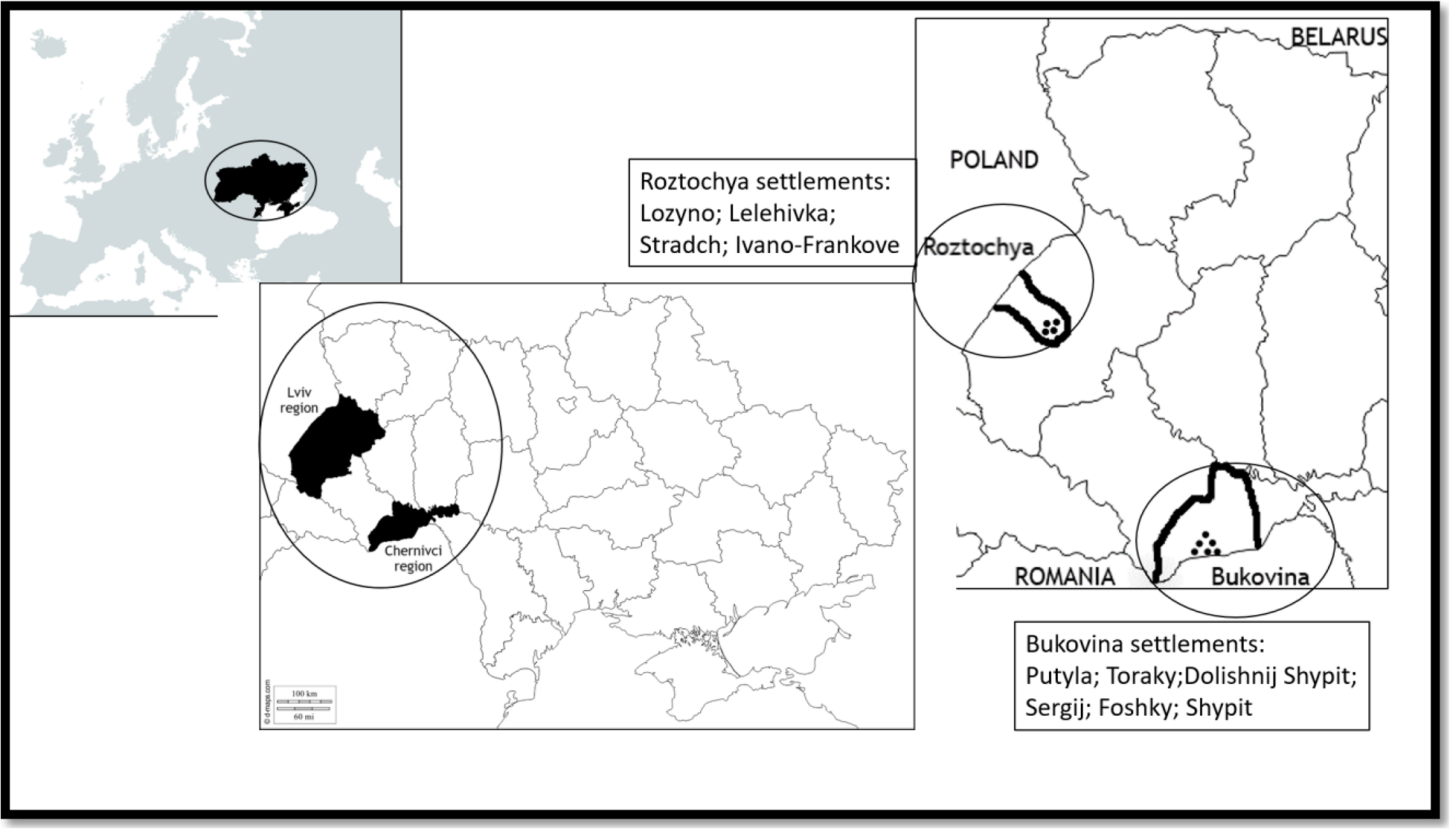
Map of the study areas. Bukovina and Roztochya in SW Ukraine

**Fig. 2:**
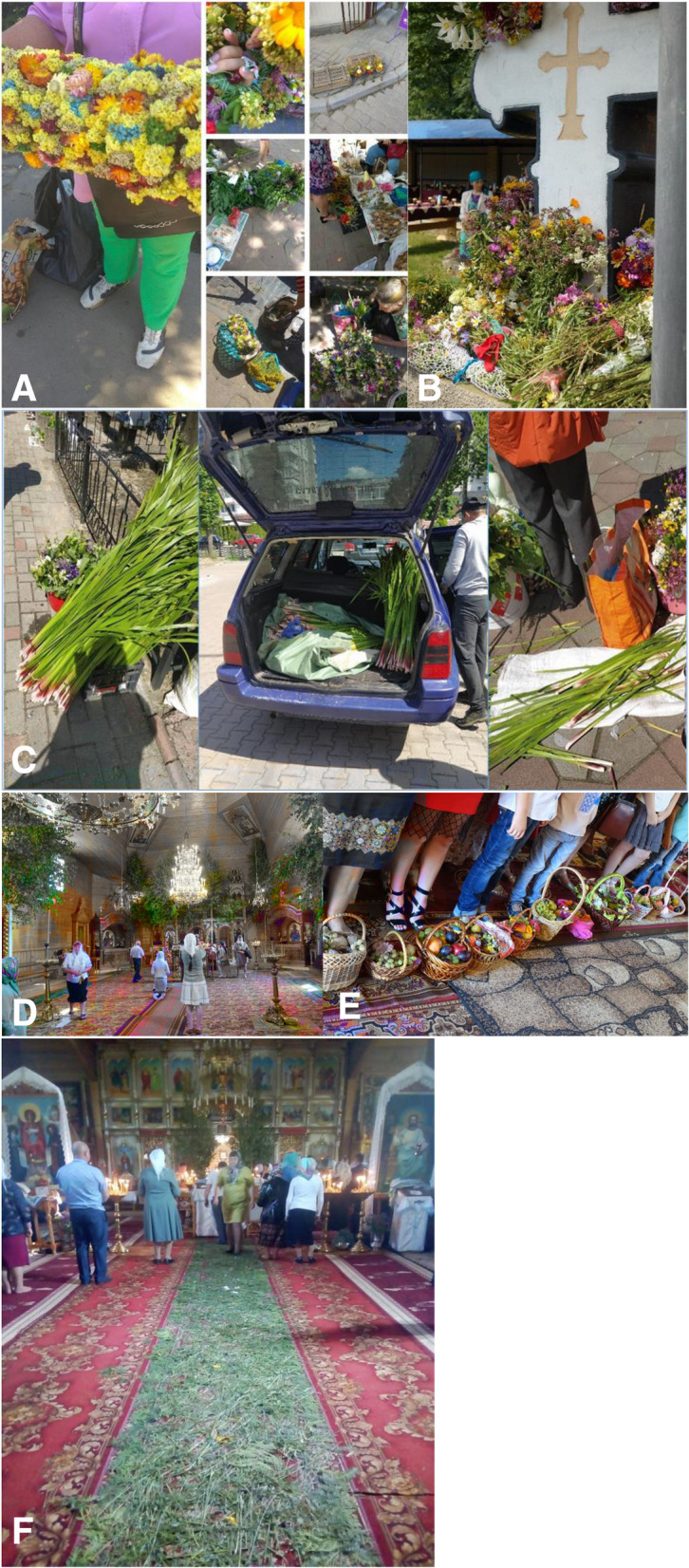
**a** The variety of plants sold in local markets before religious holidays, Roztochya region, summer 2018 and 2019; photos by N. Stryamets. **b** The blessing of plants on St. John’s Day, Bukovina region, 7^th^ of July; photo by N. Stryamets. **c** The selling of wild plants (including *Acorus calamus*) before the celebration of Pentecost, summer 2019; photos by N. Stryamets. **d** Church decorated with young *Betula* spp. trees for St. John’s day, Bukovina region, 7^th^ of July; photo by S. Nagachevskyi. **e** Fruits, honey, and poppy seeds blessed on the Spaca holiday (Apple Feast of the Savior), Roztochya region, 19^th^ of August, 2018; photo by N. Stryamets. **f** The Pentecost celebration in church in the Bukovina region, “green herb carpet”, 2019; photo by V. Bunyak

Traveling across Western Ukraine during a church holiday, one can see nicely dressed people with beautiful bouquets of wild flowers going to church. As the church is usually full, people are standing outside and observing the blessing ceremony of flowers and fruits (Figs. 1 and 2). Being an intimate human-nature interaction [1], the use of plants is an important source of inspiration and cultural identity [2]. The use of plants in rituals is a little explored corner of biocultural diversity which has developed through time within a complex socio-ecological system [3]. While local knowledge and practices are generally declining due to urbanization, a disconnection from nature, and many other factors [4], here we see the opposite―the revival of a cultural practice. The variety of plants used for bouquets by the large number of people near the church reminds us that there is a legacy of the complex history of the area (for more than 600 years the region has been part of various states with different levels of political rights and language use restrictions, e.g., the Grand Duchy of Lithuania, Polish–Lithuanian Commonwealth, Austro-Hungarian Empire, Kingdom of Romania, Second Polish Republic) [5], which is quite similar to what one can observe in neighboring Poland, where the plant richness of bouquets has been studied [6, 7]. The practice resembles that which is widely described in historical sources about these ritual practices [8]. Many scholars have highlighted the pre-Christian or pagan roots of many religious holidays in Ukraine [9–11]. In Western Ukraine, the use of plants for decoration and ornamental purposes was documented as early as the nineteenth century (see, for example, [12–14]) and has continued into the twenty-first century [9, 15, 16]. In the 1930s, in the region of Western Ukraine, the use of 85 taxa in religious ceremonies was recorded [17]; however, current practices are not just the result of an unshaken tradition. After the Soviet Union occupied Western Ukraine, church and religious celebrations were severely restricted for more than 50 years and practitioners punished [18]: churches were closed and used as storage places for grain or coal, as cultural clubs, or simply left abandon to be destroyed [19]; road crosses and chapels were demolished as they represented “visible church propaganda” [20, 21]; and priests were either killed or sent to Siberia [21]. Thus, contemporary practices are the result of a revitalization of traditional knowledge that occurred after socialism [22].

From an ethnographic perspective, the contemporary ritual use of plants in Western Ukraine has been explored in several studies (but they are mostly available only in the Ukrainian language). For example, Baglay [23] described the traditional Pentecost flowers in Western Ukraine, highlighting the apotropaic use of blessed bouquets against “bad luck” and “evil eye,” as well as storms and natural hazards, in funeral ceremonies, and for healing people and animals. Also, Kukharenko [24, 25] documented the use of plants in ritual magic for cattle. Ritual plant use in Ukraine is closely connected to that in Belarus, Poland, and Russia as state borders have changed a few times in the last century and the traditions among northern Slavs are a continuum. For example, in Poland, this subject has been well studied [26–29], and in Slovakia, Varchol [30] studied the tradition of blessing herbs among Ukrainians.

Despite this valuable ethnographic data, the actual socio-cultural impact of the interruption of these practices during the socialist era on the traditional knowledge of a community, as well as the strategies put in place for the revitalization of this knowledge, and thus the link with tradition itself that these contemporary practices express, are areas still to be explored. Moreover, there is a knowledge gap in the use of plants for cultural purposes [17]; day-to-day cultural practices following the yearly calendar with different cycles and holidays allow us to understand the role plants play not only in religious life but also in shaping identity [11, 31–33].

In order to investigate these issues, we explored the use of plants in religious rituals within two communities living in Western Ukraine: Hutsuls living in the Carpathian Mountains and the inhabitants of Roztochya living in a hilly area along the Polish border. To understand the extent to which the similar ecological conditions are reflected in their rituals and the way in which they describe the ritual components, we documented the plants used for ritual purposes among Hutsuls living in the Bukovinian Carpathians and residents of the Roztochya region, which is situated about 400 km north-west of Bukovina, close to Lviv city, where the residents have shared with Hutsuls the language of education (Ukrainian) and recent history (since Soviet occupation in 1939). To explore ritual plant uses “beyond the taxa,” we not only compared the list of plants used by the two communities and examined the origin of their practices, but we also analyzed attitudes toward the ritual use of plants and interactions with the environment in this context.

The specific objectives were:

- To document current and past uses of plants for rituals among rural inhabitants of Roztochya and Hutsuls living in the Bukovinian Carpathians;
- To detect similarities and differences regarding the ritual use of plants in the religious calendar in the two communities;
- To compare current uses reported in the two communities with historical data;
- To discuss the drivers for the selection or use of ritual plants;
- In the discussion, we will explore the possible dynamics underpinning the reactualization of traditional knowledge related to the use of plants for rituals in Western Ukraine.

## Materials and methods

### Study areas

We carried out fieldwork in the summers of 2018 and 2019 in Bukovina and Roztochya, two historical districts of SW Ukraine (Fig. 1). Both areas lie close to state borders (Romania and Poland, respectively) and are dominated by a rural population with a long and rich history of natural resource use, as well as cultural and biological diversity [33–36]. Beginning in 1199, the two regions were part of the Ukrainian Galician-Volyn Kingdom, and then from 1349 to 1774 both areas were under the control of the Kingdom of Poland and Polish-Lithuanian Commonwealth. In 1774, both areas became part of the Austro-Hungarian Empire, but in 1917 Roztochya was annexed to the Second Polish Republic and Bukovina to the Kingdom of Romania. In 1939, both areas were divided and incorporated into the Ukrainian Soviet Socialistic Republic (USSR), Roztochya as part of the Lviv region and Bukovina as part of the Chernivci region. Russian Communists in Western Ukraine forced Ukrainian people to convert from Greek Orthodox to Russian Orthodox after 1939, at which time people were also repressed and sent to Siberia. Since 1991, both areas have been part of independent Ukraine.

#### Roztochya

Roztochya (Table 1) is an ecoregion divided between Ukraine and Poland, and it is a part of the main European watershed, where several rivers going into catchments of the Black and Baltic seas start, forming an important green infrastructure corridor [39]. In 2011, the Roztochya Biosphere Reserve was designated in the area as a model site for sustainable development. In the central part of the Roztochya territory, a military training area was created in the 1940s, triggering the deportation of residents from 170 villages, and the area is still home to the largest military base in Western Ukraine [40]. The Roztochya region has been mostly settled by Ukrainians. After the Soviet Union collapsed, the chemical and mining enterprises, which employed 20,000 people, closed and this led to high unemployment in the region. Therefore, with the support of the local authorities, a “tax free” area was created to support small-scale business. Currently, locals work in the food production, chemical, building, social and forestry sectors, as well as in trade, and small-scale businesses are developed here.

**Table 1:** Ecological and socio-cultural characteristic of the study regions in 2018 (sources: [37, 38])

Characteristic/name of the region	BUKOVINA	ROZTOCHYA
Name of study area, origin	The name derives from the Ukrainian “бук” [Buk]—beech, meaning beech land	the name derives from the Ukrainian “розтікатися” [Roztikatysya], which means the flow of water in different directions
Divided into halves, 1939	Ukraine (part of Chernivci region) and Romania (part of Suceava county)	Ukraine (part of Lviv region) and Poland (part of Podkarpatske vojevodstvo)
Forest cover, %	68	40
Dominant tree species	*Fagus sylvatica*, *Picea abies*, *Abies alba*	*Fagus sylvatica*, *Pinus sylvestris*, *Quercus* spp.
Total area, km^2^	884	1548
Population density, people per km^2^	29.1	80.6
Population size and settlements	26,300 people, living in 50 villages, and 1 small town	124,000 people, living in 132 villages, 4 small towns, and 2 larger towns
Religion	58.96% Orthodox, 19.8% Greek Catholic (Orthodox), 12% Protestant, 11.2% other religions	52.6% Greek Catholic (Orthodox), 36.7% Orthodox, 11.7% other religions
Special areas	Cheremoskyi National Nature Park	Roztochya Biosphere Reserve
Language	Ukrainian, with Hutsul dialect	Ukrainian

#### Bukovina

Bukovina is a historical region (Table 1) situated in the Carpathian region on the banks of the Prut and Siret rivers. The Putyla district is considered the poorest district of the region, characterized by a negative population growth rate [37]. It is inhabited by Hutsuls, an ethnic Ukrainian group living in the Carpathian Mountains and in mountainous Bukovina, who consider themselves a “forest nation” that lives close to nature [41]. Bukovina is a rural region, with its center, the small town Putyla (3400 inhabitants), called a “village of town type.” Local residents practice natural husbandry with sheep, cows, goats, and horses. Each household has supplementary small-scale agricultural plots near the house, providing fruit (apples, pears, raspberries) and vegetables (potatoes, carrots, onions, garlic). Locals mostly work in state-financed sectors, such as schools, hospitals, and libraries. In 2011, with the aim of attracting tourism development in the region, the Cheremoskiy National Nature Park was created. After the economic crises of the 1990s, the wool-processing plant that employed more than 600 people closed down, which lead to a decrease in the number of sheep in the region. The forestry sector still provides the main employment today. Emigration to wealthier regions of Ukraine, as well as to Poland, Italy, and the Czech Republic, was identified by respondents as a major trend in the past 20 years.

### Old vs. new style calendar—a complex story

In Western Ukraine, the Old Style (Julian) calendar, which is 13 days behind the Gregorian (New Style) calendar, is used for religious holidays, while the Gregorian calendar is used for everyday life; therefore, dates are presented according to the New Style calendar (Table 2). This information is confusing because for everyday life, locals use the New Style or Gregorian calendar, as is done in the other European countries, but for religious holidays and celebrations the Old Style is still used. Officially, the whole of Ukraine adopted the new calendar on the 16^th^ of February, 1918, and Bukovina under the Kingdom of Romania changed calendars in 1919, but some ethnic groups, like the Hutsuls, have refused to change the dates of their holiday celebrations. Roztochya, as part of historical Galicia, which was under the control of the Polish–Lithuanian Commonwealth, adopted the new calendar in 1582, but as there were complicated relations between the Polish Catholic Church and the Ukrainian Orthodox Church and the Greek Catholic Church, religious celebrations have not changed to the new calendar [42, 43]. As with the Old Style calendar, term Greek Orthodox should be explained. In Ukraine, there are a number of Orthodox churches, including the Orthodox Ukrainian Church, the Moscow Orthodox Church, etc. The Greek Orthodox Church is also called the Greek Catholic Church, which celebrates all holidays according to the Old Style Calendar and is a Byzantine Rite Eastern Catholic Church in full communion with respect to the authority of Pope and the Catholic Church.

**Table 2:** Celebrated national holidays and the official Greek Catholic Church and Orthodox Church holiday (OCH) calendars (sources: [42, 43])

Date	Greek Catholic	Orthodox^b^
25^th^ of December^a^	–	–
7^th^ of January^a^	Christmas	Christmas
March-April (Changes every year)	Flowers’ Sunday (Willow Sunday, or Palm Sunday blessing *Salix* spp.)	Willow Sunday (blessing *Salix* spp.)
April–May (changes every year)^a^	Easter (blessing Paska bread and eggs)	Easter (blessing Paska bread and eggs)
May–June (changes every year)^a^	Pentecost	Pentecost
May–June (changes every year)	Corpus Christi (no information on the blessing of herbs)	–
7^th^ of July	St. John the Baptist (herb blessing not officially recognized)	St. John the Baptist (herb blessing not officially recognized)
14^th^ of August	St. Makavey (blessing of honey to give thanks for a good harvest)	St. Makavey (blessing of honey to give thanks for a good harvest)
19^th^ of August	Feast of the Transfiguration (blessing of fruits to give thanks for a good harvest)	Feast of the Transfiguration (blessing of grapes and other fruits)
28^th^ of August	Assumption of Mary	Assumption of Mary

^a^National Ukrainian holiday related to OCH

^b^In the official Orthodox calendar there are two dates—one new style, the other old style

Studies [44, 45] have demonstrated that some minority groups have not changed their habits and customs, e.g., Bukovinian Hutsuls did not change calendars in 1919, although the state and the Romanian Church did so [46]. Likewise, locals in Bukovina and Roztochya use the New Style calendar for everyday life, but for the celebration of religious holidays, they follow the Old Style calendar (both Orthodox and Greek Catholic).

There are ongoing discussions in the local mass-media, on social media and the Internet, and in the Ukrainian Orthodox and Greek Catholic churches, about changing the church calendar to the New Style [47]; however, this decision has not yet been made. The reason is that local people have a negative attitude toward this change and church authorities do not want to provoke any conflicts of interests [48].

### Data collection

This research was conducted within the framework of the ERC project “Ethnobotany of divided generations in the context of centralization” in which we aim to understand the mechanisms of change in ethnobotanical knowledge experienced by traditional societies and minority ethnic groups of Eastern Europe [49, 50]. Fieldwork was carried out between June and September 2018, with a total of 47 interviewees, 31 in Bukovina and 16 in Roztochya. We used mainly a convenient sampling method, yet sometimes, a snowball method was also used for the selection of informants. We interviewed mainly elderly individuals (average age was 61 years in both communities) permanently living in the regions. The sample comprised 70% women and 30% men in Bukovina and 75% women and 25% in Roztochya. In Bukovina, all interviewees were Orthodox Hutsuls, while in Roztochya they were Greek Catholic (97%) and Orthodox (3%) Ukrainians. The Code of Ethics of the International Society of Ethnobiology [51] was strictly followed and the study protocol was approved by the Ethical Committee of Ca’ Foscari University of Venice. Informed consent was orally received prior to each interview. Interviews were conducted in Ukrainian; however, some interviewees responded in a local Hutsul dialect or in Ukrainian with the addition of some Hutsul words. The interviews lasted from 20 to 140 min, and they were digitally recorded when permitted by the interviewee. We collected qualitative and quantitative data about various uses (food, medicinal, ritual) of local wild and cultivated plants gathered now or in the past; and the data are being used for wider research. For this study, we recorded plants used in specific religious ceremonies and practices related to the church calendar and their later application in everyday life. Specifically, we asked informants to (1) list the plants they use for religious celebrations, (2) describe the religious holidays and the way the plants are incorporated into rituals, and (3) explain the meaning of the use of plants in religious celebrations. In addition, we performed participant observation, such as conducting walks in villages, going to local markets, attending church holidays (see Fig. 2), and visiting surrounding landscapes, and analyzed the relevant local mass-media press. Interviews were semi-structured, and, whenever possible, informants were asked to point out the mentioned plants around their settlements or in the local landscape in order to harvest voucher specimens to include them in the herbarium.

We classified the used plants into three categories based on their level of wildness:

- Wild plants—taxa that grow spontaneously in the local environment without the intervention of humans (like *Betula* spp.);
- Cultivated plants—taxa that are directly managed by humans (like *Allium sativum*);
- Semi-cultivated plants—taxa growing without direct human intervention, but were once planted in gardens (like *Armoracia rusticana*).

When available, the mentioned taxa were collected and identified according to the local flora [52]; voucher specimens are deposited at the Roztochya Natural Reserve, herbarium UAV. Taxonomic identification, botanical nomenclature, and family assignments followed the Flora Europaea [53], The Plant List database [54], and the Angiosperm Phylogeny Group IV [55].

The data were assembled into an Excel database and structured in the form of detailed use-reports (DUR), which included the local plant name, Latin name, part used, preparation mode, holiday when used and additional comments. For each plant and use, the DUR was entered into the Excel database and then analyzed, taking into account the holiday in which the plants were used, mode of preparation and if it was a private (home decoration) or public (blessings in church) use. We grouped the plant taxa on the basis of the holiday in which it was used, and then a calendar of holidays and uses was constructed for both regions. Following the methodology of González-Tejero et al. [56], we calculated the Jaccard Similarity Index:

$$ \mathrm{JI}=\mathrm{C}\kern0.5em \times 100/\left(\mathrm{A}+\mathrm{B}-\mathrm{C}\right), $$

where *A* is the number of recorded species in study area 1, *B* is the number of species recorded in study area 2, and *C* is the number of species common to both study areas 1 and 2.

An index value close to 100 indicates that the two groups’ plant uses are very similar, while a value close to 0 indicates that groups are very different.

### Comparison with historical data

Historical studies of the daily livelihoods of Western Ukrainian peasants, including the use of plants for religious purposes, were published by the Shevchenko’ Scientific Society (1890–beginning of the nineteenth century), which included Ivan Franko’s ethnographical studies of Galichyna (Galicia), Bukovina, and Hutsulshchyna [12]. Detailed and comprehensive descriptions of the annual calendar, rituals, and plants used by Hutsuls [14], as well as livelihoods in the Bukovina region [13], were already provided by the end of the nineteenth century. In the past, the calendar year was connected to the cultivation and harvesting seasons, but later it was transformed through religious holidays and feasts into the religious calendar [3, 27, 57].

Ethnobotanical studies carried out by Adam Fischer in the 1930s in Western Ukraine, in which he described the plants (with their Latin names and voucher specimens) used in ceremonies and rituals, are discussed by Kujawska et al. [17]. Here, we reviewed the historical information in the Fischer dataset [17] which describes the holidays and plants used in Bukovina by Orthodox Hustuls (part of the Kingdom of Romania) [13, 14, 17] and by rural locals in the Roztochya area (part of the Lviv region which at that time was a Polish territory), in order to understand the overlap of the current nomenclature of plants used in rituals, but also to see whether the general attitude toward ritual plant use has changed in some way.

During the Soviet era, there were restrictions on celebrating religious holidays [18, 20]. There were no direct laws that forbade visiting churches, but party functionaries guarded entrances to churches (those which were not destroyed or closed) and kept a list of all those who visited, which was then discussed at Communist Party meetings. Members of the Communist Party, however, were forbidden to visit churches and to celebrate religious holidays. Family-centered holidays were replaced by worker-centered holidays [58], and religious holidays were often referred to in a satirical and sarcastic manner. According to earlier studies [59, 60], locals in both of the study regions celebrated all religious holidays in secret. However, the publications we used for comparison from the Soviet period [11, 31, 32, 61] were written by ethnographers who escaped the Soviet Union and worked in exile.

We carried out a qualitative and quantitative comparison with Fisher’s dataset, as comparison with other historical sources are more approximate since they do not provide Latin names and describe ritual uses through the local names of the plants, which cannot often be univocally interpreted [13, 14, 31, 32].

## Results

### Ritual plant use among Hutsuls in Bukovina and the inhabitants of Roztochya (Western Ukraine)

In total, 64 taxa were used by interviewees for religious purposes. We recorded 58 plant taxa belonging to 36 families in Roztochya and 28 plant taxa belonging to 22 families in Bukovina that were used in seven religious celebrations (Table 3). We documented 118 DUR of plants in Bukovina and 238 DUR of plants in Roztochya. Our data show that wild plants were used more than cultivated species in rituals; however, the situation was not the same in the two regions. In both areas, locals used two more wild taxa than cultivated ones.

**Table 3:** Use of plants for religious rituals in Ukrainian case study areas

Taxa/Voucher specimen	Local name/transcribed name format	Wild/Cultivated/Semi-cultivated	Plant part (s) used	Use	Holiday	Frequency of use in Bukovina, BN = 31	Frequency of use in Roztochya, RN = 16
*Acer* spp. (Sapindaceae) SF019	ясен, клен/ yasen, klen	W	Fresh branches	Decoration of entry doors, garages, gates and barns	Pentecost	4	1
		W	Whole young tree	Decoration of yards	Pentecost		
*Achillea millefolium* L. (Asteraceae) SF020	деревій/ derevij	W	Aerial parts	Bouquets	Pentecost St. John’s Day	1	1
*Acorus calamus* L. (Acoraceae)	шавар, май/shavar, maj	W	Aerial parts	Decoration of yards and roads	Pentecost		4
*Alchemilla vulgaris* aggr. (Rosaceae)	приворотень/pryvoroten	W	Leaves	Bouquets	Pentecost		1
*Allium sativum* L. (Amaryllidaceae) SF021, SF061	часник, чеснок/chasnyk, chesnok	C	Aerial parts and tubers	Basket decoration	Easter	9	
				Bouquets	Feast of the Transfiguration		13
*Anemone nemorosa* L. (Ranunculaceae)	анемона біла/anemona bila	W	Aerial parts	Bouquets	Palm Sunday		1
*Armoracia rusticana* P.Gaertn., B.Mey. & Scherb. SF008 (Brassicaceae)	Хрін, хрен, хреню hrin, hren, hrenyu	SC	Roots	Basket decoration	Easter	19	13
*Artemisia abaensis* Y.R.Ling & S.Y.Zhao (Asteraceae) SF227	полин, полинь, полин гіркий, полин трава/ polyn, polyn`, polyn girkyj, polyn trava	W	Aerial parts	Bouquets	Feast of the Transfiguration, Makoviya		2
*Asarum europaeum* L. (Aristolochiaceae) SF200	копитняк/ kopytnyak	W	Leaves	Wreaths	Corpus Christi		5
*Beta vulgaris L.*( Amaranthaceae)	буряк/buryak	С	Roots	Basket decoration	Easter	1	
*Betula* spp. (Betulaceae) NB049	береза/bereza	W	Fresh branches	Decoration of entry doors, garages and barns	Pentecost	5	3
			Whole young tree	Decoration of yards	Pentecost		
*Buxus sempervirens* L. (Buxaceae) SF023	буршпан, гуршпан/ burshpan, gurshpan	C	Branches	Bouquets, basket decoration and candle decoration	Easter, Palm Sunday		7
*Campanula* spp. (Campanulaceae)	дзвоники/ dzvonyky	W	Aerial parts	Bouquets	Pentecost, Feast of the Transfiguration	1	2
*Centaurea* spp. (Asteraceae)	васильки/vasyl`ky	W	Aerial parts	Bouquets	Feast of the Transfiguration	1	
*Cyanus segetum* Hill (Asteraceae)	волошка/voloshka	W	Aerial parts	Bouquets	Pentecost		1
*Dianthus barbatus* L. (Caryophyllaceae)	гвоздика городня/gvozdyka gorodnya	C	Aerial parts	Wreaths	Corpus Christi		1
*Dianthus* spp. (Caryophyllaceae)	гвоздика/gvozdyka	C	Aerial parts	Bouquets	Pentecost		3
*Dryopteris filix-mas* (L.) Schott (Dryopteridaceae) SF036	папороть/paporot`	W	Aerial parts	Bouquets	Pentecost		1
*Fraxinus excelsior* L. (Oleaceae) SF018	ясінь/yasin’	W	Aerial parts	Branches decoration of yards and homes	Pentecost	2	
*Fragaria × ananassa* (Duchesne ex Weston) Duchesne ex Rozier (Rosaceae) SF037	полуниця/polunycya	C	Aerial parts and fruits	Wreaths	Corpus Christi		2
*Fragaria vesca* L. (Rosaceae) SF038	суниця/sunycya	W	Aerial parts and fruits	Wreaths	Corpus Christi		4
*Hedera helix* L. (Araliaceae) SF039	плющ/plyushh	W	Aerial parts	Wreaths	Corpus Christi		1
*Juglans regia *L. (Juglandaceae) SF006	горіх/gorih	C	Fruits	Table decorations	Christmas	3	1
*Hypericum perforatum *L. (Hypericaceae) SF024	звіробій/zvirobij	W	Aerial parts	Bouquets	Saint John’s Day	4	
*Iris* spp. (Iridaceae)	півники дикі/pivnyky dyki	W	Aerial parts	Bouquets	Pentecost		1
*Leucanthemum* spp. (Asteraceae) SF064	ромашка/romashka	W	Aerial parts	Bouquets	Pentecost (R) Feast of the Transfiguration (B)	1	4
*Malus domestica* Borkh. (Rosaceae) SF040	яблука/yabluka	C	Fruits	Basket filling, bouquets	Feast of the Transfiguration (B,R)	10	12
*Matricaria chamomilla* L. (Asteraceae) SF041	румянок/rumyanok	W	Aerial parts	Wreaths Bouquets	Corpus Christi (R) St. John’s Day	2	1
*Mentha* spp. (Lamiaceae) SF042	мята/myata	C	Aerial parts	Bouquets	Pentecost		4
*Myrtus communis* L. (Myrtaceae)	мірта/ mirta	C	Small branches	Basket decoration	Easter		1
*Onopordum acanthium L.*? *Cirsium* spp.	будак/budak, страхополох/strahopoloh, чортополох/chortopoloh	W	Aerial parts	Bouquets	Feast of the Transfiguration	1	3
*Paeonia* spp. (Peoniaceae) SF063	півонія/pivoniya	C	Aerial parts	Bouquets	Pentecost		2
*Papaver* spp. incl. *Papaver rhoeas* L., *Papaver somniferum* L. (Papaveraceae) SF050	мак/mak	W, C	Seedpods	Bouquets, basket filling	Feast of the Transfiguration (R,B) Easter (B)	11	14
*Persicaria bistorta* (L.) Samp. (Polygonaceae)	ракові шийки/rakovi shyjky	W	Aerial parts	Wreaths	Corpus Christi		1
*Picea abies* L. (Pinaceae) SF043	ялинка/yalynka	W	Whole young tree, branches	Home decoration	Christmas	1	1
*Polygonatum* spp. (Asparagaceae)	купина / kupyna	W	Aerial parts	Wreaths	Corpus Christi		1
*Prunus cerasifera* L. (Rosaceae) SF013	алича/alycha	C	Fruits	Basket filling	Feast of the Transfiguration	1	1
*Prunus avium* (L.) L. (Rosaceae) SF045	черешня/chereshnya	C	Fruits	Basket filling	Feast of the Transfiguration		1
*Prunus cerasus* L. (Rosaceae) NB198	вишня/vyshnya	C	Fruits	Basket filling	Feast of the Transfiguration	1	3
*Prunus domestica* L. (Rosaceae)	сливки/slyvky, сливи/slyvy	C	Fruits	Basket filling	Feast of the Transfiguration	1	9
*Pyrus communis* L. (Rosaceae) SF017	грушки/grushky	C	Fruits	Basket filling	Feast of the Transfiguration	1	10
*Rosa* spp. incl. *Rosa rugosa* L. (Rosaceae)	рожа/rozha	SC	Aerial parts	Wreaths	Corpus Christi		5
*Rubus ideaus* L. (Rosaceae) SF051	малина/malyna	C	Fruits	Basket filling	Feast of Transfiguration		2
*Salix* spp. (Salicaceae) SF052, SF062	верба, verba, баська, bas`ka, лоза, loza, шутка, shutka, бичка, bychka	W	Young branches	Bouquets, branches	Palm Sunday	22	14
*Sambucus nigra* L. (Adoxaceae)	хобза/hobza	W	Young branches	Decoration of yards	Pentecost	1	
*Scilla bifolia* L. (Asparagaceae)	анемона голуба, anemona goluba, проліска/proliska	W	Aerial parts	Bouquets	Palm Sunday		1
*Secale cereale* L. (Poaceae)	жито/zhyto	C	Spikes	Bouquets	Christmas	1	1
*Sedum acre* L. (Crassulaceae)	очиток/ochytok, розкідник/rozkidnyk	W	Aerial parts	Wreaths	Corpus Christi		7
*Senecio vulgaris* L. (Asteraceae)	жовтозілля/zhovtozillya	W	Aerial parts	Wreaths	Corpus Christi		1
*Solanum lycopersicum* L. (Solanaceae)	помідори, томати/pomidory, tomaty	C	Fruits	Basket filling	Feast of the Transfiguration (L)	1	2
*Symphytum officinale* L. (Boraginaceae) NB190	живокість/zhyvokist`	W	Aerial parts	Wreaths	Corpus Christi		1
*Tagetes* spp. (Asteraceae)	чорнобривці/chornobryvci	C	Aerial parts	Bouquets	Feast of the Transfiguration		2
*Quercus* spp. (Fagaceae) SF063	дуб/dub	W	Fresh branches	Decoration of yards	Pentecost		1
*Thymus* spp. (Lamiaceae) NB186	чебрець пахучий/ chebrecz` paxuchyj	W	Aerial parts	Wreaths	Corpus Christi, St. John’s Day	1	1
*Tilia* spp. (Malvaceae) SF053	липа/lypa	W	Fresh branches	Decoration of entry doors, garages and barns	Pentecost	2	5
*Tilia* spp. (Malvaceae) SF053 *Triticum aestivum* L. (Poaceae)	липа/lypa пшениця/pshenycya	W C	Whole young tree	Decoration of yards	Pentecost	1	1
			Spikes	Bouquets			
*Triticum aestivum* L. (Poaceae) *Vaccinium myrtillus* L. (Ericaceae) SF049	пшениця/pshenycya чорниця/ chornycya	C W	Spikes Aerial parts with berries	Bouquets Wreaths	Makoveya		12
					Corpus Christi		2
*Vinca minor* L. (Apocynaceae) SF010	барвінок/barvinok	W	Aerial parts	Wreaths	Corpus Christi		10
*Vitis vinifera* L. (Vitaceae) SF001	виноград/vynograd	C	Fruits	Basket filling	Feast of the Transfiguration	5	5
Poaceae SF060	злаки/ zlaky	C	Spikes	Bouquets, ‘Didukh’	Christmas (B,R)	2	4
Poaceae SF060 Hay^a^	злаки/zlaky сіно/sino	C W	Spikes Aerial parts	Bouquets, ‘Didukh’ Table decorations	Makoveya (B,R)		4 8
					Christmas (B,R)	3	
Herbs^a^	зілля / zillya	W	Aerial parts	Bouquets	Pentecost (R) St. John’s Day (B)	8	5

^a^Hay and Herbs are named for bouquets and table decorations in general, meaning dried or fresh varieties of wild plants, which interviewees did not want to explain or there was no specific need to explain in the opinion of locals (e.g., “*We collect all beautiful herbs.*” or “*At the meadow you collect all that you like.*”). *BN* Bukovina number of interviews, *RN* Roztochya number of interviews, *B* Bukovina, *R* Roztochya

More than 50% of respondents in Roztochya also mentioned hay as a table decoration for Christmas dinner. Another 30% in Roztochya and 23% in Bukovina only named green herbs for blessing ceremonies, not wanting to discuss further the specific taxa used. The respondents mentioned general bouquets without specifying any names, simply saying “herbs,” “any herbs,” and “just beautiful green herbs.” Some respondents highlighted that they should be “medicinal herbs.”

*Salix* spp. was the most used taxon, being named by 68% of Hutsuls and 81% of interviewees in Roztochya. In Roztochya, *Armoracia rusticana* (75%), *Allium sativum* (62%), *Papaver* spp. (75%), *Triticum aestivum* (68%), *Malus domestica* (75%), and *Pyrus* spp. (60%) were named by a majority of interviewees. *Vinca minor* was named by 50% of interviewees in Roztochya, and also shown by the historical data to be the most used taxa in Western Ukraine [17]. *Sedum acre* and *Prunus domestica* were used by 37% and 56% of interviewees, respectively. One third of the interviewees in Roztochya named *Vitis vinifera*, *Asarum europaeum*, *Allium cepa*, *Tilia* spp., and *Rosa* spp., including *Rosa rugosa*, as used in various celebrations. Twenty-five percent of interviewees used *Dianthus* spp., *Mentha* spp., *Leucanthemum* spp., *Fragaria vesca*, and *Acorus calamus*.

Hutsul interviewees named the following taxa: *Armoracia rusticana* (62%), *Papaver* spp. (36%), *Malus domestica* (35%), *Allium sativum* (29%), *Vitis vinifera* (16%)*, Betula* spp. (16%), and *Hypericum perforatum* and *Acer* spp. (more than 13%).

**Fig. 3:**
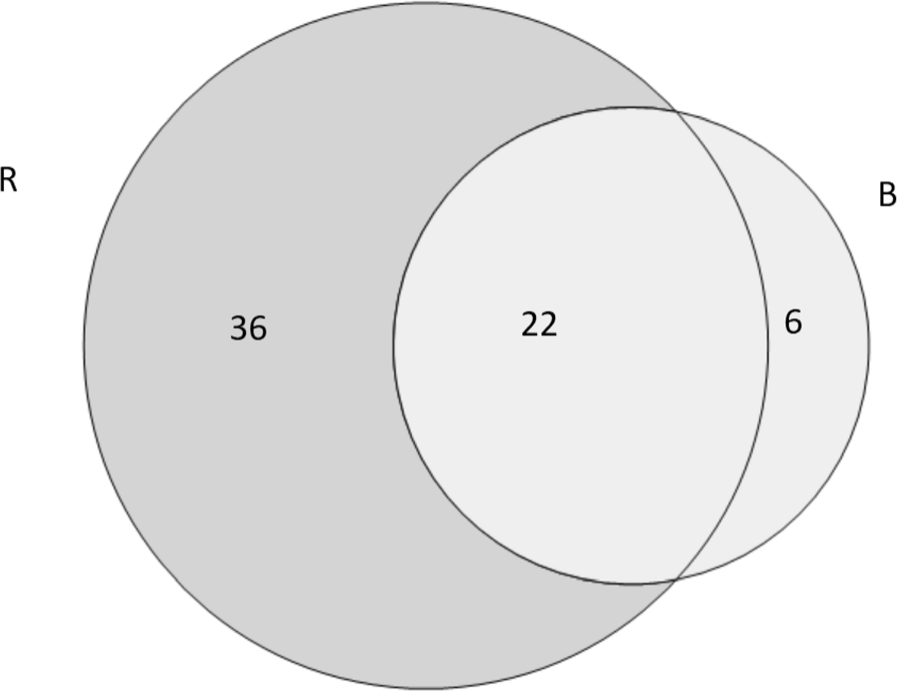
Venn diagram of taxa used in rituals in the two study areas (R: Roztochya, B: Bukovina)

Comparing the use of plants in rituals and celebrations, 22 taxa were used in both study areas (Fig. 3).

**Fig. 4:**
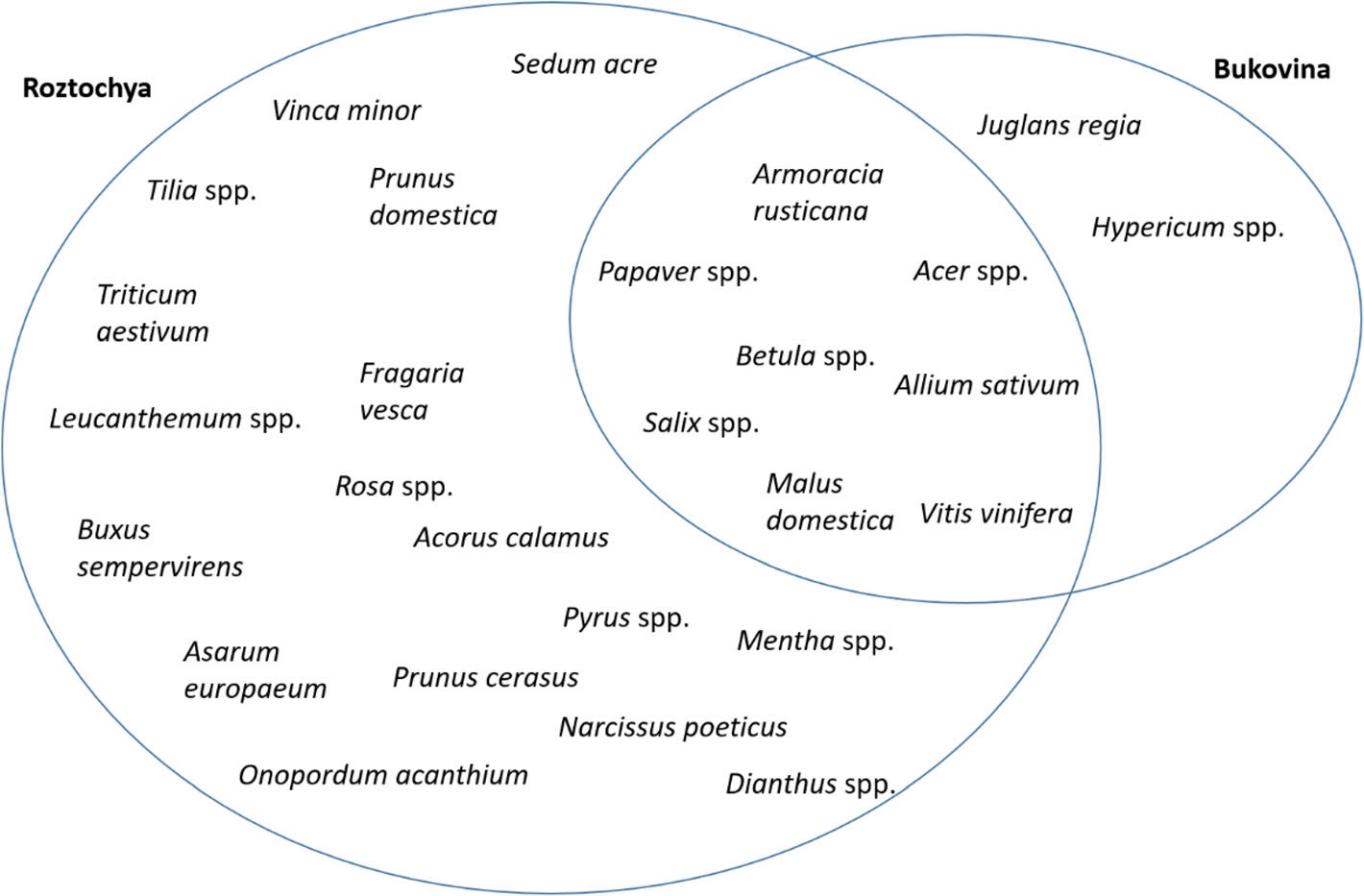
Plant taxa named by more than 10% of interviewees used for holidays and in rituals in the two study areas

In addition to *Salix* spp., the most used taxa common to both communities were *Armoracia rusticana*, *Papaver* spp., *Malus domestica*, *Allium sativum*, *Vitis vinifera, Betula* spp., indicating the dominance of cultivated taxa. In addition to a comparison of all data, we compared only those taxa that were named by more than 10% of interviewees in order to harmonize the data (Fig. 4).

### Are the uses the same? Similarities and differences in ritual plant use

We recorded 37 wild plant taxa, 25 cultivated plant taxa and 2 semi-cultivated plant taxa used in the two Ukrainian study areas (Table 3). In Roztochya, wild plant taxa dominated with 22 used taxa, followed by 20 cultivated taxa, and only two semi-cultivated taxa. In comparison, Bukovinian Hutsuls named 16 wild taxa, 14 cultivated taxa, and one semi-cultivated taxon. Thus, in both areas, wild taxa predominated, but cultivated taxa were also quite important.

The Jaccard similarity index for the use of plants for ritual purposes was 34.38, which indicates that the uses are quite different between the two regional groups.

**Fig. 5:**
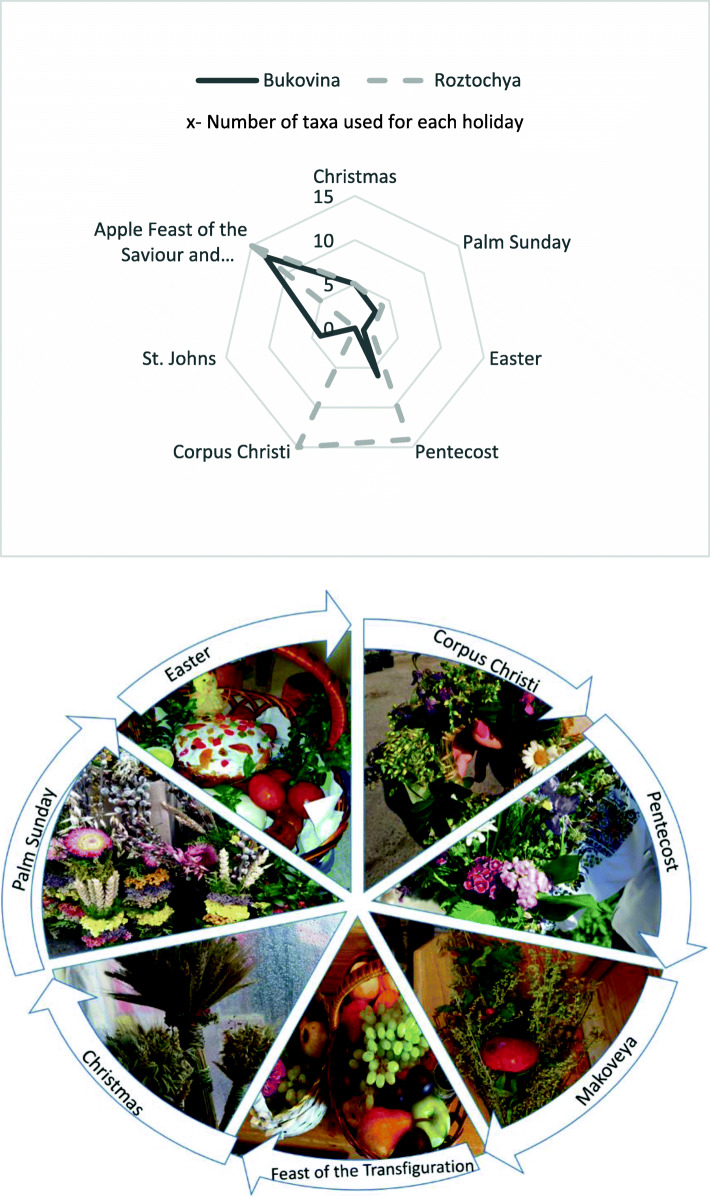
Diversity of holidays in which the use of different number of plants is incorporated (in the top) and year cycle of holidays (in the bottom). Photos by N. Stryamets, 2018-2019.

The distribution of the number of taxa used for holidays (Fig. 5) clearly shows that the summer-based holidays are the richest in terms of taxa used in both study areas. In both areas, interviewees reported the most taxa used for celebration of the Apple Feast of the Saviour and Makoviya. In the Roztochya area, the Corpus Christi and Pentecost holidays were also rich with 15 and 14 plant taxa used, respectively. In comparison, in Bukovina, Hutsuls reported only six taxa used for Pentecost. The winter holidays, like Christmas, were much less represented with plant uses.

**Fig. 6:**
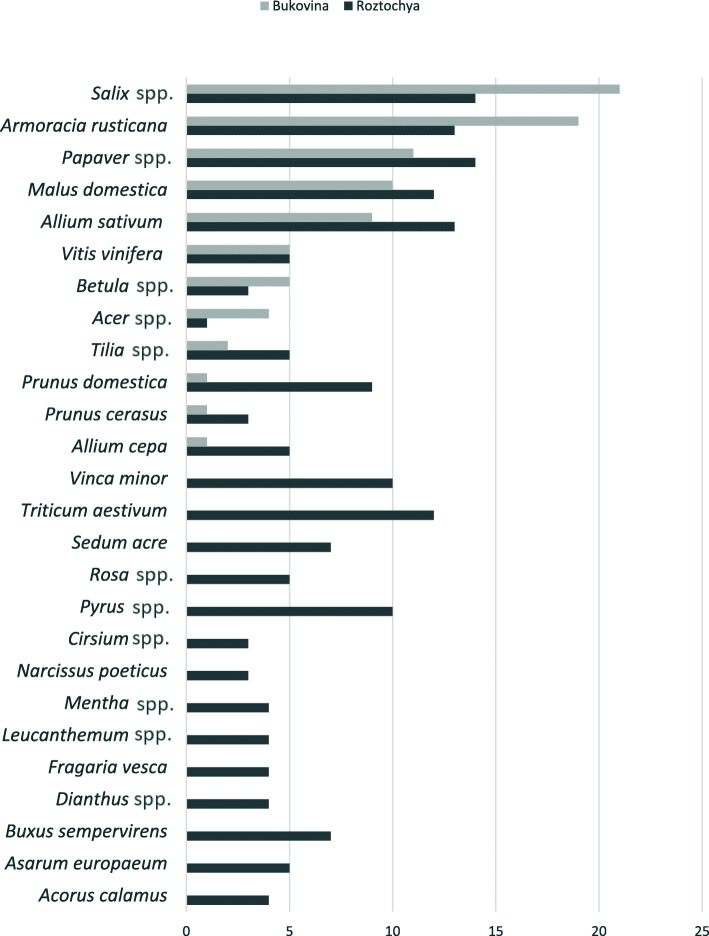
Most used taxa in the two study areas

Among the most used taxa (Fig. 6), *Salix* spp. and *Armoracia rusticana* are predominant in both study areas. Seven taxa were equally popular and named in both areas.

### The use of ritual plants in the religious calendar

On the basis of the interviews, we recorded 7 holidays that incorporated plants used in rituals; these were mainly Christian holidays, but some celebrations have pagan roots (e.g., Kupala).

**Fig. 7:**
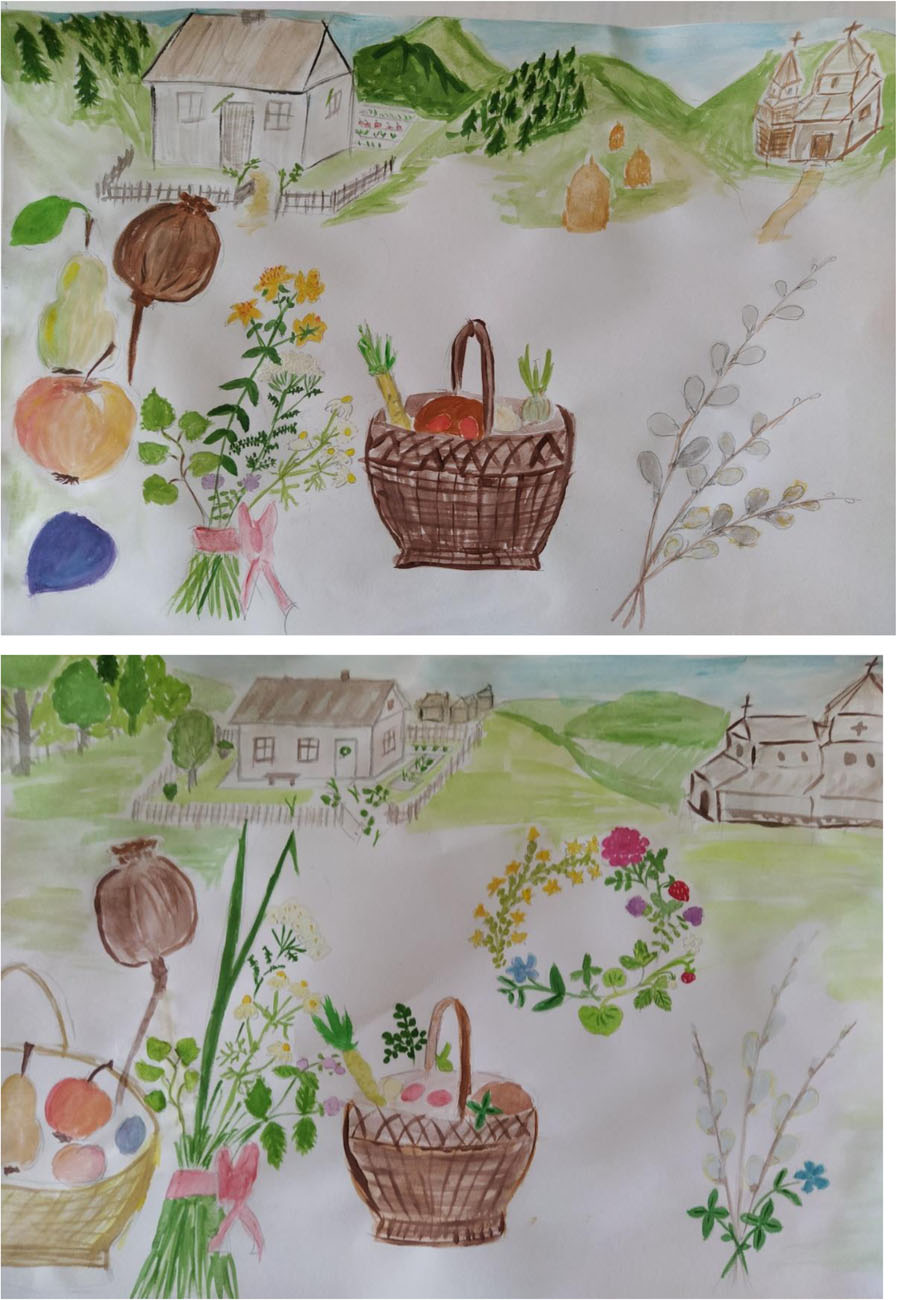
Drawings of rural households and plants used in church blessings and for home decoration in Bukovina (above) and Roztochya (below). Drawings by S. Stryamets

In having the same calendar, but not having the same rituals described by the official church, there were two main differences in the celebrations named by interviewees: in Roztochya, bouquets of various herbs were blessed on Pentecost (May–June) while in Bukovina bouquets were blessed on St. John’s Day (July 7^th^); and wreaths were blessed on Corpus Christi (June) in Roztochya, whereas in Bukovina there was no such practice (Fig. 7).

Comparison of our results with the historical data from Bukovina [13] revealed the disappearance of the celebration of two holidays with plants incorporated into their rituals, including the celebration of St. George’s Day (May 6^th^) with the burning of herbs and the decoration of gates with grass and herbs to protect against witches. The Fischer data showed that in the Roztochya region, locals used plant bouquets for blessing on the Assumption of Mary (August 28^th^). While such a practice is still widespread in Poland [7], a single interviewee from Roztochya mentioned the blessing of bouquets on Assumption Day in the Ivano-Frankivsk region of Ukraine.

#### Christmas–Різдво–[Rizdvo]–7th of January, celebrating the birth of Jesus (Orthodox and Greek Catholic)

Historically, for the celebration of Christmas, Christmas trees were decorated or bouquets designed and arranged. The “diduh” was a bouquet of wheat which was supposed to be harvested in your own field in August and kept until Christmas. Nowadays, the respondents buy “diduh” at the market, or they have started to abandon this tradition. The historical data mentions the use of “diduh” as a symbol of wealth and a good harvest that has pre-Christian roots [14, 32, 41].

In both study areas, the dining table for the Holy Supper (traditional Lenten meal on Christmas Eve) is decorated with dried hay, then garlic gloves, walnuts, and poppy seeds, and then a white tablecloth. “*We cook 12 dishes for Christmas Eve*, *and we decorate the table with hay*,” explained a retired Hutsul woman. The Christmas tree was rarely mentioned (only one interviewee in each region named it).

#### Palm Sunday–Willow Sunday–Вербна Неділя, Бичкова Неділя [Verbna Nedilia, Bychkova Nedilia]—1 week before Easter, celebrating when Jesus entered Jerusalem

*Salix* spp. branches were used equally in both areas as the main taxon for Palm or Willow Sunday; names that derive from the use of *Salix* spp. which is referred to as “Вербна неділя” [Verbna nedilia] in Roztochya and “Бичкова неділя” [Bychkova nedilia] in the Hutsul dialect in Bukovina. In some churches, the priest or the priest’s assistant was responsible for harvesting a large amount of *Salix* spp. branches for the people that would come to church, and each would get a branch of *Salix*; this custom was widespread among the Bukovinian interviewees. “*We take a willow branch and go to Romania to have it blessed*,” explained a Hutsul woman born in 1969, as the nearest church was across the border in Romania. *Salix* spp. decorated with ribbon was only popular among Hutsuls—“*They bless willow in church, we get it there, the priest gives it to everyone*,” explained a female Hutsul interviewee born in 1942, while in Roztochya a variety of dried herbs were used. Some interviewees in Roztochya named *Buxus sempervirens* as a decoration for *Salix* spp. bouquets, as well as spring forest flowers, e.g., *Anemone nemorosa* and *Primula veris.* In our case studies, the saying “*Not me*, *but the willow tree*, *is beating you because in one week Easter is coming*,” can be explained by the fact that the willow protects against evil and aids in “*beating out the devil*.” The willow tree has a double meaning in Ukrainian folk beliefs with evil being present in old trees and providing protection against evil when the tree is young [11]. According to Hutsul folk beliefs, when Jesus was met by children, they welcomed Him with willow branches and for that reason this custom was kept, as documented by Kaindl [13]. The historical data confirmed the use of “bychka” [*Salix* spp.] for blessing on Willow Sunday in Bukovina as early as 120 years ago [13]. Surprisingly, the Fischer dataset has no record of *Salix* spp.

#### Easter–Паска, Великдень–[Paska, Velykden]–the time of celebration changes, first Sunday following the first new moon after the spring equinox, celebrating the resurrection of Jesus (April–May)

An essential practice on Easter, which was mentioned in both communities, was to have priests bless baskets containing different foods: meat, a special Easter bread called Paska [Паска], painted eggs, cheese and butter, sausages, and smoked meat. Hutsul interviewees explained that it is “*obligatory*” to have their food blessed on Easter. They decorated baskets with young garlic leaves and horseradish roots with young green aerial parts. In Roztochya, baskets for Easter were decorated with *Buxus sempervirens* as well as all “*spring flowers*.” The aerial parts of garlic were named as obligatory in Bukovina, but not in Roztochya. Horseradish roots were named in both study areas as an important component in Easter celebrations both as a ritual food (beetroot salad with horseradish) and for ritual decorations. It was explained that blessed horseradish was used with eggs and in seasoning meat. The historical data shows the blessing of food for Easter in both areas, naming colored eggs called “писанки” or “pysanky” as obligatory. A female Hutsul interviewee, born in 1969, highlighted that “*Here people don*’*t paint pysanka anymore*; *only elders know how to do it*; *when they all pass away*, *no one will know this practice*.”

The historical data demonstrate rather strong resilience of the food blessing practice on Easter by Hutsuls [13, 14] and in the Lviv region [32], with *Armoracia rusticana* as one of the requisite products for blessing throughout the twentieth century. Recent ethnographical studies in the Chernivci region also reveal the use of *Armoracia rusticana* and *Allium sativum* for the Easter blessing [62].

#### Pentecost–Green Sunday–Трійця–Зелені свята [Triitsia-Zeleni sviata]—50^th^ day after Easter, commemorating the descent of the Holy Spirit upon the Apostles (May–June)

**Fig. 8:**
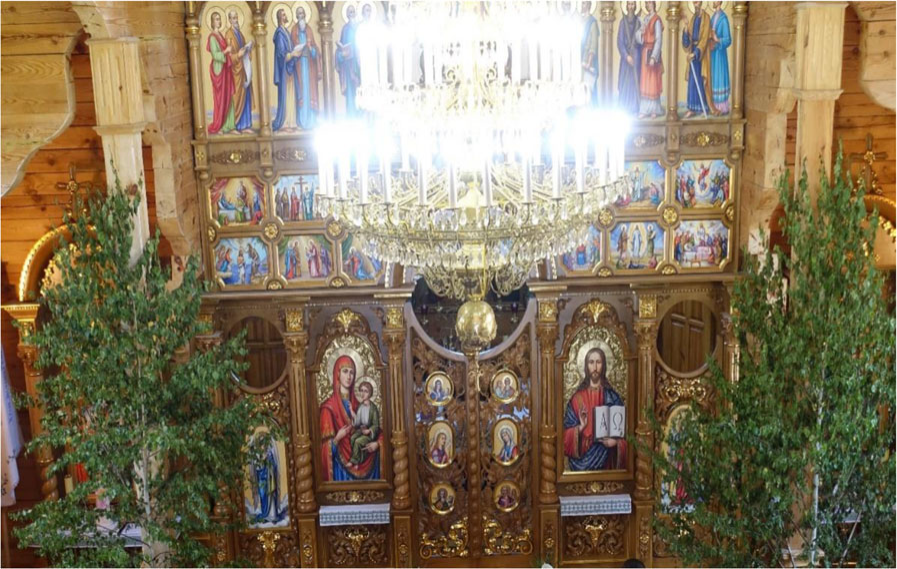
Church decorated with young *Betula* spp. trees in Roztochya for Pentecost, 2019; photo N. Stryamets

The local name of Pentecost is “Zeleni Svyata” [зелені свята] or Green Holiday, because everything is so green and beautiful [23]. According to our field data, in both regions, the tradition of decorating houses with young branches on Pentecost has never been interrupted. During the lifetime of our interviewees, in both Roztochya and Bukovina, houses, stables, and gates were still decorated with branches or the whole young tree of *Betula* spp., *Tilia* spp., and *Acer* spp. (Fig. 2d). Bouquets of different forest plants were blessed during this feast. Yards and churches were often decorated with young *Betula* spp. trees (Fig. 8).

Kaindl [13] mentioned the decoration of yards and churches in Bukovina. The historical data reveal the decoration of churches, yards, and houses with tree branches in both areas [23, 63]. Voropai [31] and Kylymnyk [32] explained that Pentecostal blessing of herbs has pagan roots. The name of the holiday in Ukrainian, Green Holiday, derives from a pre-Christian celebration of trees and nature. *Quercus* and *Tilia* trees have been considered to have been incorporated into celebrations and rituals during pre-Christian times [57].

Yards, roads, gates, and fences are decorated with the aerial parts of *Acorus calamus*, and before Pentecost they are sold in markets everywhere (Fig. 2c). The interviewees could not explain the significance of many decorations and used expressions like “*my grandmom used to do so*,” and “*it is because the spring is green*, *everything is green*, *and so we decorate yards*. *The holiday is called* ‘*Green Holiday*’” (woman, born in 1965, Roztochya). A Hutsul woman, born in 1960, explained that she used the “*yellow bog plant called* ‘*zovti slipaky*’” [probably *Acorus calamus*, but she could not provide a more specific description nor a voucher specimen, or *Iris pseudacorus*, which has very similar leaves to *Acorus calamus*] for fence decoration to protect against all evil, which could not enter the yard; this was done for Green Sunday and St. George’s Day. She was the only interviewee who discussed this holiday and the use of this plant; however, the historical data [13] mention this use of *Acorus calamus*.

The practice of blessing bouquets was exclusive to the Roztochya area. Our Roztochya interviewees stated that they either collect the flowers themselves or buy them from the local market. They named some specific plants, such as *Mentha* spp., *Dianthus* spp., *Alchemilla vulgaris*, and *Paeonia* spp., but also just mentioned the beautiful and colorful wild and cultivated flowers. According to Hutsul folk beliefs [13, 17, 61], flower bouquets are considered to have strong medicinal properties and they are used for apotropaic purposes. Our interviewers still remember using plants from Pentecostal bouquets for healing: “*In the past people used these bouquets when cows were sick*. *They knew what herbs should be in the bouquets*. *Nowadays we use herbs from the garden*, *like mint and other flowers and herbs*.” At the same time, the tradition of using such bouquets for funeral rituals was stronger and still preserved today: “*We still use these bouquets for making a pillow for a deceased person; these bouquets are put into the coffin*” (woman, born in 1959, Roztochya).

Historically, aromatic herbs (like *Mentha* spp.) were used to decorate the house and yard to protect against evil forces [31, 32]. *Acorus calamus* was used against bad water spirits and this practice is still widely used in Roztochya (Fig. 2c). This practice of decorating the house and yard was also observed during Soviet times as it was not forbidden to decorate the home [31]. The trees historically used for yard decoration [11, 31, 61], such as *Quercus* spp., *Tilia* spp., and *Acer* spp., are the same as those used in both areas today. In Bukovina, Hutsuls decorated churches with carpets of herbs (see Fig. 2f), which has been described as a historical practice [31]. Yards and fences were decorated with the branches of different types of trees (oak, birch, acer, and linden) in both study areas (Figs. 2d and 8).

#### Corpus Christi–Боже тіло [Bozhe tilo]—60^th^ day after Easter (May–June)

In Roztochya, wreaths made out of a variety of plants were used for blessing on this holiday (Fig. 2a). Our interviewees explained that each household must have three wreaths. The obligatory plants included *Fragaria* spp., *Vaccinium* spp., and other forest species. Also, one respondent pointed out that “*One has to know how to make proper wreaths*, *so it is better to buy them*. *My grandmother always made three wreaths*, *one was made from rozhidnuk* [*Sedum acre*], *and two were made from other herbs*. *Then those wreaths were kept in the house as protection against evil*” (woman, born in 1959, Roztochya). “*My mom told me that it should be a separate wreath from rozhidnyk* [*Sedum acre*],” highlighted another woman born in 1979. This is in line with the Fischer data, where *Sedum acre* was named for the Corpus Christi blessing. “*All medicinal herbs are used*,” explained a woman born in 1963. “*The wreath should be near the entrance door for the whole year*,” highlighted a woman (born in 1963) from the Roztochya area.

In Bukovina, such wreath blessings were not reported, as Orthodox Christians do not celebrate this holiday. Historically, in the areas around Lviv, this practice was named by Fischer, but it was not observed in Bukovina based on Kaindl [13].

#### Kupala–St. John’s–Івана Купала–Івана зілльового [Ivana Kupala–Ivana Zillovoho]—7^th^ of July, Saint John’s Day

In the Bukovina region, herbal bouquets were blessed at Orthodox churches on the 7^th^ of July, yet this was not reported in the Roztochya district. Different forest and meadow plants were used (Fig. 2b). The church was decorated with *Betula* trees and occasionally other plants (Fig. 2d). Attention was given to plants that have medicinal properties, as according to interviewees this holiday takes place at the proper time to harvest medicinal plants. “*All herbs need to be collected before the Ivana holiday*, *then blessed*, *then used as medicine or just kept in the home to protect from all evil*. *But one should know which herbs to use*,” highlighted a Hustul man born in 1980. “*All the* [*medicinal*] *herbs should be collected before the Ivana holiday*, *and bathing with those herbs is good*,” explained a Hutsul man born in 1950. “*We blessed zillya* [*herbs*] *on the Ivana Zilliyovogo holiday*,” explained a Hutsul woman born in 1942, and “*the blessed herbs are given to cattle*,” highlighted a middle-aged Hutsul woman. Most of the interviewees referred to this holiday as “*Ivana Zillyovogo*,” “*Kupala*,” or just “*Ivana*,” no one called it St. John the Baptist Day. “*Starting with this herb*, *everything needs to be collected before the Ivana holiday*, *before Ivana all herbs need to be harvested*,” explained a Hutsul man born in 1950. The collection of medicinal plants after St. John’s Day was not allowed [32]. Remarkably, 23% of respondents mentioned bouquets in general, not specifying any plant taxa, but rather “*just beautiful green herbs*” for blessing.

According to popular beliefs, which were documented by Kainld [13], gate decoration with herbs was used to protect against witches. Different “zillya” [medicinal herbs] were blessed on this day and later used for medicinal purposes. Kylumnyk [32] and Hilarion [57] suggested that before the advent of Christianity, Ukrainians used to celebrate a number of holidays connected to the seasons of the year and the harvest. They proposed that the Kupala holiday (*Купальська ніч*) celebrated at midsummer was then incorporated into the Saint John’s Day holiday, but the ritual of herb collection and that medicinal properties of those herbs retained the best qualities left from that time.

The blessing of herbal bouquets was described by Voropay [31] and Kylymnyk [32]; however, both of them detailed the pre-Christian roots of this celebration. The specific plant here was St. John’s herb (*Hypericum perforatum*), which after blessing was used for healing humans and cattle [31, 61]. In Bukovina, *Hypericum perforatum* blessed on St. John’s Day was named by 11% of interviewees. The medicinal plant was then stored at home behind an icon of the Saint [31], which is in line with the description of one interviewee.

There has also been discussion, lead by Kononenko [9], that the re-vitalization of the Ivana Kupala holiday could be of Soviet origin, as in the 1960s some calendar holidays and rituals were re-introduced, especially in Central Ukraine, and this could be the case in Bukovina.

#### The Makoviya or Makaveya–Маковея-Маковія–[Makoveia–Makoviia]—14^th^ of August, Honey Feast of the Savior

**Fig. 9:**
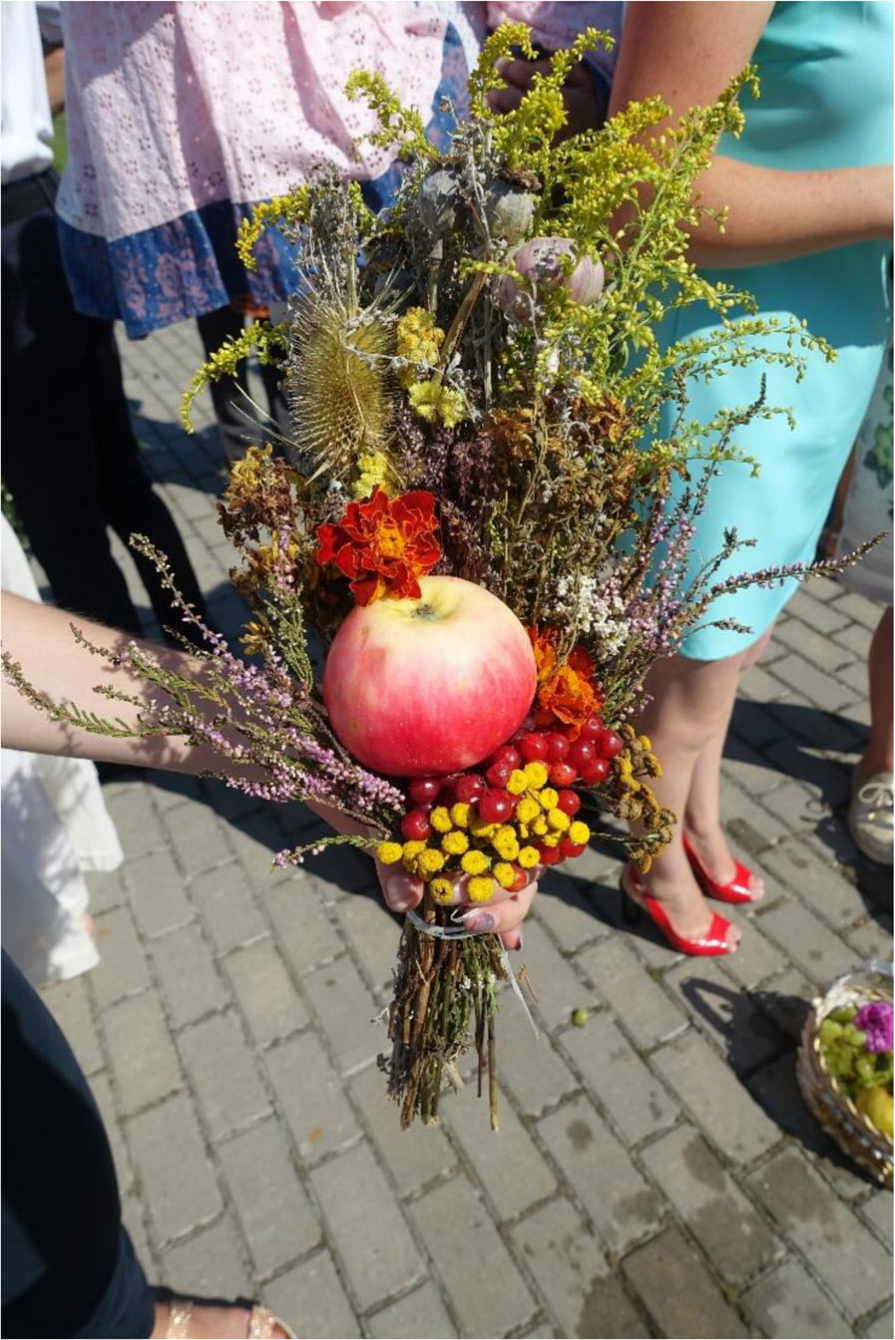
Blessed bouquet on the Makoviya holiday, 19^th^ of August, 2018; photo by N. Stryamets.

According folk belief, the name Makoviya derives from that of poppy seeds: “mak” in both Ukrainian and Russian. However, church authorities have argued that it is the name of Saint Makkavei and has nothing to do with poppy seeds (Fig. 9).

Poppy seeds with garlic gloves and *Arthemisia absinthium*, as well as different herbs, were named as parts of the baskets for blessing in both study areas. Respondents in Roztochya identified *Arthemisia absinthium* and *Cirsium* spp. as the main herbs for this holiday. Honey was also named as a product to be blessed. A Hutsul woman born in 1965 said, “*We bless honey and poppy seeds on Makoviya*.” The blessed herbs from the bouquets were used for the fumigation of children and cattle explained a middle-aged woman from the Roztochya area. Most of the interviewees declared that both Makoviya and the Transfiguration (see below) were the fruit blessing holidays, and baskets were often blessed on a single day (as there are only 5 days between these holidays).

Historical sources describe the blessing of honey, water, and poppy seeds on this holiday, which is referred to as “First Savior–Першого Спаса [Pershogo Spasa].” *Calendula officinalis*, *Tagetes paluta*, and *Ocimum basilicum* were among the herbs documented by Voropay [31].

#### Apple Feast of the Savior–Яблучний Спас–Спаса [Yabluchnyi Spas–Spasa]—19^th^ of August, celebrating the Feast of the Transfiguration

Garden fruits as well as poppy seeds and garlic gloves are used for blessing in both study areas. Apples are considered the main fruits of the holiday. The flowers of *Tagetes* spp. were used for the decoration of fruit baskets. Our interviewees explained that in the past only fruit from home gardens were used, but nowadays purchased fruit is also included: “*Nowadays even oranges are blessed*.” The baskets with fruits as well as bouquets with fruits and herbs were named as equally important for blessing on this holiday (Fig. 2e). “*We bless poppy seeds and herbs and honey and grapes and apples on the Transfiguration holiday*,” highlighted a Hutsul woman born in 1965.

In Bukovina, Kaindl [13] explained that Hutsuls did not eat fruit from their own gardens before this holiday. Only after the fruits were blessed in church on Transfiguration Day were Hutsuls permitted to consume them. A Hutsul woman, born in 1984, explained that now also apples can only be eaten after they are blessed on the Feast of the Transfiguration: “*Before the Spasa holiday one cannot eat apples. Apples and honey are blessed on that holiday*.”

### Overlap of current ritual plants with historical sources

**Fig. 10:**
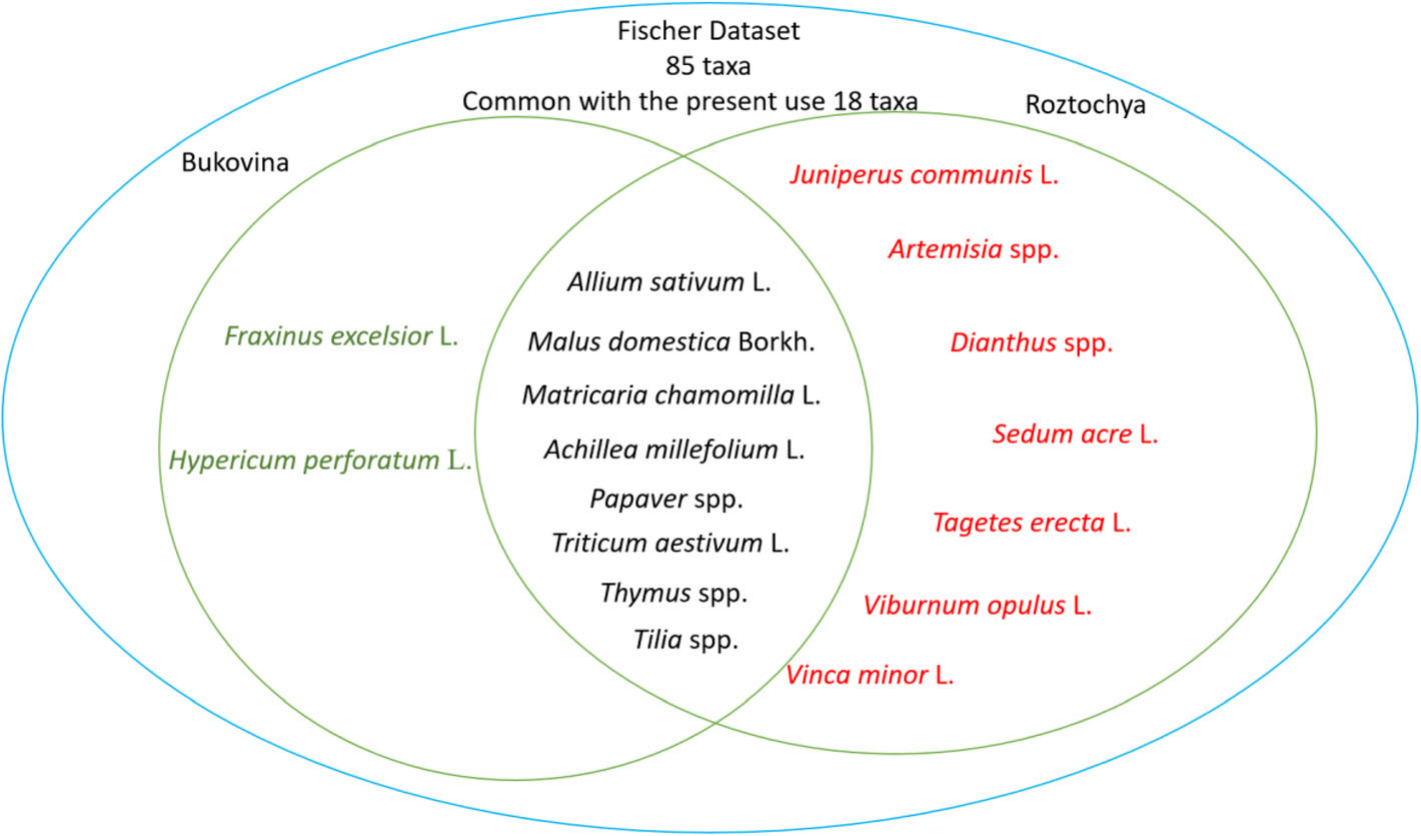
Common taxa used in rituals in Roztochya and Bukovina compare to the Fischer dataset (overlap shows taxa mentioned in all three datasets)

The list of plants used in ceremonies and rituals recorded by Adam Fischer in the 1930s, in the territory of Western Ukraine, contained 85 taxa [17], which is 28 more than were identify in the current study; so we can assume that the diversity of uses today has changed compare to 100 years ago, and one of the factors that influenced the decrease in the variety of taxa used was the prohibition period. But according field results, some traditions were not interrupted. The diversity of past and present uses of plant taxa is shown in Fig. 10. The data provided by Kujawska [17] that *Vinca minor* was the most used taxon for hair decoration by brides is in line with the statement of a retired female interviewee in Roztochya: “*In the past every bride had a wreath with barvinok* [*Vinca minor*], *but nowadays this custom is gone.*” Blessed herbs were burned during storms, against thunder, which is also consistent with the explanation regarding the importance of blessed herbs today given by interviewees from both Bukovina and Roztochya. Out of a combined 64 taxa used in Roztochya and Bukovina, only 18 taxa were shared with the dataset of 85 taxa used in the rituals documented by Fischer [17] (Fig. 10). Among the abundant plants which are no longer used are cultivars that were grown as decorative plants (*Phlox* spp., *Ruta graveolens* L., *Helianthus annuus* L., *Helianthus tuberosus* L., *Myrtus communis* L., *Nigella damascena* L., *Calendula officinalis* L., *Malva sylvestris* L.), aromatic plants (*Salvia officinalis* L., *Origanum vulgare* L. *Carum carvi* L.), and ruderal plants (*Amaranthus hybridus*, *Plantago media*, *Urtica* spp.). However, the use of *Origanum vulgare* and *Calendula officinalis* were witnessed at church blessing ceremonies during participatory observation, but were not named by interviewees.

Eight taxa were commonly used in Roztochya and Bukovina and also listed in the Fischer dataset as plants used in rituals. Those taxa are equally distributed among cultivated (*Malus domestica*, *Triticum aestivum*, *Papaver* spp., *Allium sativum*) and wild plants (*Achillea millefolium*, *Matricaria chamomilla*, *Thymus* spp., *Tilia* spp.) (Fig. 10).

**Fig. 11:**
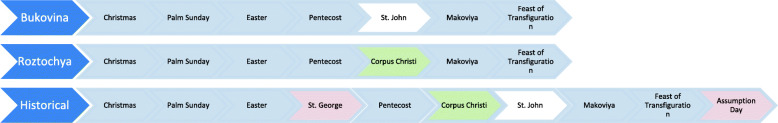
The holidays incorporating the use of plants in rituals in Bukovina and Roztochya today and in the past—historical data

The decoration of yards for Willow Sunday and Pentecost are the same, but in Bukovina the decoration of houses and gates takes place on St. John’s Day (Fig. 11), while in Roztochya wreaths blessed on Corpus Christi are put close to entrance doors for apotropaic purposes. Only *Salix* branches are blessed in Bukovina while in Roztochya a whole bouquet of willows is used. The study by Piotr Köhler [64] on sepulchral plants and plants blessed in the nineteenth century from the Rostafiński questionnaire also documented one use of *Salix* spp. for Easter celebrations (but not for Palm Sunday). The blessing of medicinal herbs takes place on St. John’s Day in Bukovina, while in Roztochya it is bouquets on Pentecost. Fruits in both areas are blessed on the Transfiguration holiday (Fig. 11). For the Roztochya territory, the study by Kujawska [65], based on the study by Moszyński (1929), described the use of plants for the decoration of house roofs with an apotropaic purpose, but the holiday has changed as in the 1930s they were used on St. John’s Day, while today they are used on Pentecost.

**Fig. 12:**
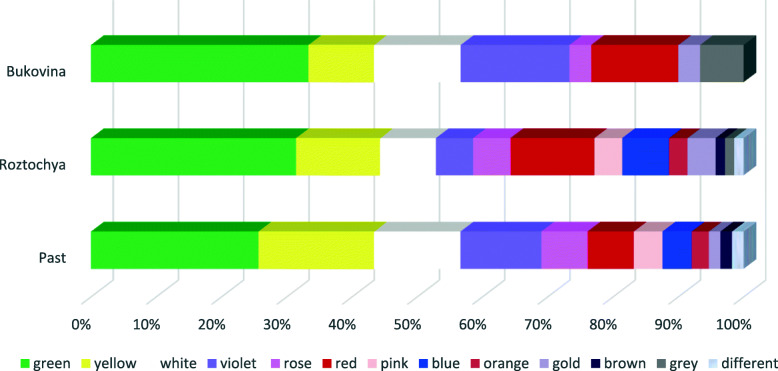
Palette of colors used in the two study areas today and in the past

The dominant color in all three datasets was green, followed by yellow and white in Roztochya and in the past, and white and purple in Bukovina (Fig. 12). Nowadays, in both areas, the color red is used more than in the past. The inhabitants of Roztochya mentioned the use of the color blue, and likewise it was used in the past, but it is not used in Bukovina.

In the past, wild plants dominated with 67 used taxa, compare to just 22 taxa in Roztochya and 16 taxa in Bukovina. Cultivated plants in the Fischer dataset were represented by 27 taxa, while 20 taxa were used by the inhabitants of Roztochya and 14 taxa were used by Bukovinian Hutsuls. Semi-cultivated plants were represented by two used taxa in Roztochya, and one taxon both in Bukovina and in the past.

### Possible drivers of the selection and use of ritual plants

From our research, it appears that changes in plant use hinge on a plurality of interconnected factors. First of all, the selection of a plant is linked to the dominant aesthetic of the time. The choice of “only beautiful herbs,” as the informants often explained, should be framed within the religious meaning of the ritual itself and the implicit use of flowers as symbols of the festival and the offering within the festival. In this respect, the aesthetic choice is understood as an expression of a devotional act.

However, as Bourdieu [66] points out, taste is affected by sociocultural factors and changes over time in relationship to the actual interaction among different groups in a society. Thus, the change should be read, first of all, as the combined effect of the application of traditional knowledge passed on over generations (“My grandmother told me to use those herbs” was a recurrent motive in the interviews), in a context of transformation of ethnobotanical practice marked by the introduction of more cultivated plants. Our shows that the use of wild taxa was more prevalent in the past compared to that of today in both study areas. Over generations, this change affects aesthetic criteria and can explain the fact that nowadays people tend to use cultivated taxa and spend less time collecting wild plants. As one of the interviewees from Roztochya claimed “we have become lazier, we don’t collect wild herbs anymore.”

In parallel with this, we can also observe how interviewees had a preference for medical plants. This particular choice expresses a form of associative thinking that is widely attested to in European folklore [8, 67, 68]. In particular, the choice is based on a sympathetic relationship between the beneficial effects of the plants and the redeeming message of the Christian rituals.

## Discussion

### Knowledge and believes

Despite strong prohibition during the Soviet era, the use of the plants in rituals and celebrations survived in Western Ukraine. Our results show that personal childhood memories, as well as cultural practices and the church’s influence in the study areas, play a key role in local practices involving the use of wild flowers in religious rituals. As the respondents explained, they do this ritual because our grandparents taught them to do so, which is in line with Kozholyanko [45].

For the majority of interviewees, the custom of blessing plants and decorating homes is a family tradition: “*My parents told me to do so.*” It was a common statement (21% of interviewee) that the custom is transferred from older generations. In Bukovina, interviewees stated: “*my grandma used to do so*.” This was a common response when an interviewee could not provide a clear explanation as to why they used different herbs in religious holidays and celebrations. They explained that it was what their parents and grandparents used to do and “*everybody does so*.” They stated that it was part of the culture and the history of plant use. “*We have become lazy*, *we know what to bless*, *but don*’*t like to do it anymore*,” expressed a 52-year-old woman in Roztochya. Also, “*we can buy all bouquets*, *so there is no need to collect herbs ourselves*,” explained a woman born in 1979. Therefore, the declining ritual use of plants was mentioned as a general trend in the context of urbanization and depopulation of rural areas and a disconnection with the cultural identity of Hutsuls.

### Source of authority

Our results show that besides the existing knowledge in communities, the church and priests as sources of authority have an influence on the plants used in rituals. In Roztochya, a woman born in 1949 highlighted that “the *priest says to do so*.” The church plays the role of promoter of rituals and therefore of plant use in rituals and celebrations because after the Liturgy the priest explains that they will bless bouquets or baskets. However, only а few interviewees mentioned the church as a source of knowledge. One example provided by an interviewee involves an Orthodox priest explaining to the local people that the “*wreaths for Corpus Christi are a Polish tradition*, *not Ukrainian*” (e.g., not an Orthodox but a Catholic ritual). Another female interviewee explained that “the *priest says it* [*blessed bouquet*] *is protective against evil*, *you should keep the bouquets in your home*.”

“We can buy it”

A couple of respondents (both in Roztochya) explained that they “*have become lazy*,” “*we can buy wreaths and bouquets*,” and “*children are not interested*.” Yet at the same time they highlighted “*it is our tradition, it is so nice to use herbs*.”

“*Nowadays one can buy everything and you don*’*t need to go and collect herbs yourself or make wreaths yourself*,” explained one retired male interviewee from Roztochya. Participatory observation in locals markets before each holiday revealed the great variety of bouquets, wreaths, and ornamental plants available for purchase (Fig. 2).

Historically, the local rural population was poor and church calendars were the only means of observing time [14]. The seasonal and nature-connected holidays and rituals were used as a yearly calendar for many centuries [32]. Those traditional practices and beliefs are still present in both study areas.

### Past and present

Revitalization of historical practices involving the use of plants in rituals in both study areas resulted in slight differences in holidays and modes of plant use. Nowadays in Bukovina, St. John’s Day incorporates the blessing of bouquets, while in Roztochya herbs are blessed on Pentecost and wreaths are blessed on Corpus Christi. Compare to the historical data, the blessing of bouquets on Assumption Day is no longer practiced in either Roztochya or Bukovina. The St. George’s Day holiday historically involved the blessing of fields with holy water for a good harvest [11] and the use of plants for blessing the grazing of cattle for the first time. One interviewee explained that in the past blessed *Salix* branches were used for blessing in the first time cattle grazing ceremony. In Roztochya, the blessing of bouquets on the Assumption of Mary was used in the past (based on Kujawska, [17]), but not today. Interviewees from Roztochya also mentioned the blessing of bouquets on Assumption Day in the Ivano-Frankivsk region of Ukraine, while earlier studies have shown that this practice is still present in celebrations among Lemkos and Ukrainians living in Poland [27, 29, 30]. The connection between the two areas, and their influence on each other, can be seen in the fact that Assumption Day and the blessing of bouquets is on the 15^th^ of August in Poland, while in Ukraine people bless bouquets to celebrate the Makoviya holiday on the 14^th^ of August.

The increasing use of the color red in Roztochya and Bukovina today may be the result of fashion and the desire to “show off”; while Beldiy [69] explained that red is a symbol of men and blue a symbol of women; however, Kolosova [70] highlighted that the color red is a symbol of blood in Christianity. The colors blue and white symbolize the Virgin Mary [70], while yellow is associated with medicinal properties (for the liver and kidneys) and St. John’s Day [70].

There is a strong belief in both study areas that blessed herbs will provide a security function (e.g., protect against disaster or bad luck). The Orthodox Church explained the blessing of herbs and the decoration of yards as: “*the old church used herbs and trees for the decoration of churches*, *the new church keeps this tradition as it is a symbol of Jesus Christ*” (web page, 2019). There have been two main discussion threads in the local mass-media and on the Internet: the first centers on the Old and New Style calendars, which mostly starts with the recognition by Constantinople of the Ukrainian Orthodox Church (independent from Russia), while the second concerns the view that the blessing of fruits and herbs is a pagan tradition, which has no significance in church. Priests of the Orthodox Church have also commented on the blessing of herbs and fruits, pointing out that this is a pagan tradition [71] and more likely a “thanking for the harvest” than a Christian ritual [71]. The Church does not give any importance to poppy seeds, but pagans did [71].

Informants in both study areas mostly responded that blessed herbs “*protect against evil*.” One interviewee stated: “*In my house I have had Salix bouquets for 5 years* (*one bouquet from the Palm Sunday of each year*), *as it is forbidden to throw them away*; *one can only burn them*.” The apotropaic use of blessed plants was named as important for livelihoods in both areas. “*You can eat one* ‘*cat*’ [*the bud of Salix spp*.] *after it is blessed and you will not have sore throat pain for the whole year*. *Also*, *when it is a gentle storm you can burn it and the storm will end*,” explained a Hutsul woman born in 1962. “*If you put a small branch of blessed Salix on your entrance door*, *it will protect your home from bad luck*,” highlighted another Hutsul woman. “*The bouquet blessed on St. John*’*s is put behind the icon in the house to protect against all evil*,” explained a Hutsul woman born in1965.

### Market influence or competitiveness?

Interviewees in both areas explained that today they not only use fruit from their own home gardens for the blessing of baskets, but they also buy peaches, apricots, grapes, and other fruits to “show off.” Likewise, for Palm Sunday, instead of just *Salix* (as is still used in Bukovina), they use bouquets made with a variety of herbs. Respondents explained that earlier the priest or his assistant prepared *Salix* branches for Palm Sunday and then at the end of the service the branches were given to each person present, but nowadays this custom has changed and people bring their own *Salix* bouquets to church. “*My mom explained to me that it has to be willow*, *that is why it is called Willow Sunday*, *but now it has changed to bouquets*,” declared one male interviewee born in 1959. In Bukovina, this custom is still present in some villages, such as Putyla, Ploska, Kyselyci, and Shepit. Also, in Bukovina, tomatoes were named for Easter basket decoration, as they are expensive in early spring and this is a way to “show off” or prove your secure financial status.

At local markets, we observed a variety of plants sold in bouquets and wreaths before each holiday (Fig. 2a). The prices ranged between 25 and 50 UAH (approximately 1–2 EUR) for one bouquet. Bouquets of *Salix* spp., with a diversity of dried plants, were also sold, and interviewees explained that in the past only willow branches were blessed, but now many other “beautiful” herbs are added; however, Bukovinians used only the branches of willow trees.

Our results demonstrate that both public (blessed at church) and private (decoration of homes and yards, apotropaic use (fumigation of children, keeping the dried herbs at home to protect the house)) uses of plants were present in both regions. Public uses were influenced by marketing (e.g., *Salix* bouquets instead of *Salix* branches alone) and also by competition between neighbors (who has the nicest basket or the most beautiful bouquet): “*Instead of bringing fruits from their own gardens for blessing*, *people buy fruits*, *even exotic ones*” (woman, born in 1965); “*Now it is easy*—*you can buy everything*, *willow bouquets are so pretty and you can buy them everywhere*” (man, born in 1959, Roztochya). Therefore, private uses of plants, such as hay for table decoration, Christmas “*Diduh*,” and yard and fence decoration for a variety of holidays, were named by respondents.

### The dynamic of the reactualization of traditional knowledge

#### Identity through plant use?

This research reveals the overall persistence and revitalization of the folkloric use of flowers and plants as a ritual element within the Christian religiosity of the study areas. Local practices can be viewed within the broader framework of the agrarian religiosity of the wider region that extends from western Russia down to Ukraine [8]; and, in this respects, an analysis of the present-day rituals [72] recorded in Bukovina and Roztochya suggests a double continuity, both in structure, which is the actual use of floral and herbal elements in popular rituality, and phenomenology, which is the actual selection of specific botanic varieties and species. This evidence opens up questions on the actual role played by communism in the ritual transformation of communities, as well as on the hermeneutic meaning of the current ethnobotanical knowledge that the ritual practices express. Here, we have discussed the possible influence of official holidays in Ukraine on the cultural use of plants (Table 2. Therefore, there is no connection between official holidays in Ukraine and the celebration of holidays that incorporate the use of plants (3 holidays compared to 7 celebrated with plants used in rituals)).

The cultural policies of the Soviet state led to a limitation in public religious practices, and also of Christian churches [73]. Animated by a secular intent, the restriction involved in particular the most folkloric aspects of the rituals, such as the devotional use of plants [74].

At the same time, almost paradoxically, folkloric aspects were removed from the public space on a grassroots level, while they were made the subject of in-depth scholarly studies aimed at identifying, cataloguing and preserving all aspects of Slavic heritage [75]. On a national level, new archives and museums about local folklore were opened and extended research was funded [76], while at the local level, traditional practices were pushed into the private space. This research highlights the effects of this relegation in the two regions, Bukovina and Roztochya.

Even the Soviet propaganda that, in 1935, started to promote the celebration of the New Year with Christmas trees [77] did not leave detectable traces in private religious rituals of Hutsuls. Our results are in line with Kylymnyk [32] who did not mentioned Christmas trees as an important element of celebrations in Western Ukraine. Prof. Ivan Franko [12] highlighted that Hutsuls used hay on the table as well as “Diduh,” and rich people in towns adopted the new tradition of celebrating with Christmas trees.

At the same time, the restrictions of the communist period did not result in a complete erosion of local practices. Rather than extinction, the marginalization created interstitial, domestic spaces where traditional knowledge survived and was passed on to new generations. Our research suggests that after the fall of the USSR these marginal and domestic spaces turned into a reservoir into which communities later tapped to retrieve and reactualize their traditions. This finding resonates with the ethnographic evidence concerning another form of folk religiosity, that of shamanism, specifically in Siberia [76]. In the Tuva Republic, on the border of Mongolia, Tuvians, an indigenous community of the region, were the subject of severe persecution in order to eradicate shamanistic practices during the communist era [77]. While shamanism was removed from the public space, its practice survived underground. At the end of communism, shamanism was at the center of public attention [78, 79]. The religious practice was not just legitimized, but became the subject of a wider process of recovery, restoration, promotion, and innovation [80] twinned with new political demands for cultural and socioeconomic recognition, as well as a rising search for identity by new generations of Tuvians [81]. Similarly, this research shows that in Bukovina and Roztochya traditional practices have regained publicity and centrality within religious and community practices. As in the case of Tuvians, the communist period led to the partial loss of ritual knowledge and its contemporary reinvention. This is dictated by the erosion of community memory [82], as well as the adaptation of traditional practices to new environmental and socioeconomic dynamics developed after communism. Current practices have been affected by this socio-economic transformation, when it became possible to visit churches and perform rituals and blessings, as well as affordable to buy bouquets to be blessed. However, our research suggests that locals respond to the transformation of agricultural practices, foodways, and the use of the environment, as well as to the impact of senilization and emigration.

Folklore studies in Europe suggest that when traditional knowledge is recovered after a long period of cultural marginalization, the revival is the result of a combination of knowledge passed on through generations in the domestic sphere and knowledge found in official and academic sources, such as academic research, museums, and other relevant cultural institutions [22, 83–86]. In this respect, people in Bukovina and Roztochya looked to clergymen to receive feedback, verify their knowledge, and then confirm its appropriateness. This process offers insight into how the revival of tradition modifies and repolarizes the relationship of a community with their natural surroundings; a process that places formal religious knowledge at the center of a process of reappropriation of the environment.

#### Reactualization of traditional knowledge

Our results show a shift from wild to cultivated plants being used in blessing ceremonies, which may be the result of changing lifestyles and “lazier people.” The other important point is that the interviewees tended to mention “just beautiful herbs” as being used, not specifying the names of individual plants—probably due to a disconnect with nature and thus not knowing plant names. The color palette of plants used in blessing ceremonies also changed to include more red and blue and less yellow. The regional differences in plant use may perhaps be the result of Polish influence (e.g., Corpus Christi celebrations [28]) and local fashion, competitiveness, and market influence (use more beautiful bouquets instead of just *Salix* branches). The revitalized traditional ecological knowledge (TEK) in the two study areas has transformed differently, with the inclusion of more diverse uses in Roztochya, which might be due to Polish influence and the close proximity of Lviv City with its variety of markets, where bouquets are sold before religious holidays.

While traditional ecological knowledge is “a cumulative body of knowledge and beliefs handled down through generations by cultural transmission about the relationship of living beings with one another and their environment” [87], we can conclude that the use of flowers and plants in Bukovina and Roztochya is the last remaining expression of the local TEK. Following Wiersum [88], religious beliefs [89], as well as culturally valuable practices and rituals, represent interactions between humans and biodiversity, whose complex connections are formed by history [90], cultural and ethnic belonging, and traditions in the region. In particular, what this research has highlighted is that contemporary practices are the combination of residual TEK and new information gathered from different contingent sources. This knowledge, therefore, does not only speak about the ancestral past of the community, but also sheds light on its deep and more recent history, the challenges it experienced, and the strategies it embraced in order to survive and maintain its social practices. Thus, these practices tell us about the existing relationship with the environment, its change over time, as well as folk religiosity and the legacy of communism in the community.

## Conclusions

Contemporary practices concerning the use of plants in Christian rituals in Bukovina and Roztochya in Western Ukraine can be contextualized in the broader phenomenon of the revitalization of traditional environmental knowledge and practices that have characterized Europe over the past 30 years and in particular Eastern Europe after socialism. The current religious use of plants is to a certain extent the revitalization of historical rituals supported by various internal (knowledge from older generations) and external (church authorities and fashion in the region) drivers. The current study reveals a persistence of knowledge related to ritual plants as an expression of local identity, which despite their prohibition during Soviet times, and several foreign occupations of Western Ukraine, resulted in a strengthening of language and tradition (including the use of plants for religious purposes) as an expression of local identity. However, in this revitalization, the number of taxa used in the two regions has decreased, in particular among Hutsuls. The revitalization expresses a deep transformation in the body of folkloric knowledge. It has been affected on the one hand by the marginalization of practices during the Soviet period, and on the other by the recombination that has occurred in the past 30 years, during which residual domestic knowledge combined with other information coming from scholarly and educated sources. Moreover, it also reflects the profound socioeconomic transformation that affected the community rewriting their relationship with the environment. In this respect, what we see today can be considered an expression and revitalization of traditional ecological knowledge of ever-changing communities.

## Data Availability

All data are available in this publication.

## Abbreviations

*TEK*: *Traditional ecological knowledge*
*LEK*: *Local ecological knowledge*
*DUR*: *Detailed use report*
R: People in Ukrainian Roztochya
*B*: *Hutsuls in Ukrainian Bukovina*
